# General Index to the British and Foreign Medical Review

**Published:** 1848

**Authors:** 


### GENERAL INDEX

TO THE

### BRITISH AND FOREIGN MEDICAL REVIEW.

| | VOL. | PAGE |
|---|---|---|
| ABANO, Pietro di, biography of | xix | 533 |
| ABBÉ RUCELLERI, history of | v | 406 |
| Abdomen, collections of pus in the | iv | 48 |
| diseases of the | ix | 473 |
| on bandaging in lying-in women | iii | 267 |
| on diagnosis in tenderness of | iii | 262 |
| on physical signs of the | iv | 290-1 |
| on wounds penetrating | xxiii | 2 |
| pendulous, discourse on | xiv | 215 |
| penetrating wounds of | xxi | 421 |
| syncope from blows on | xviii | 216 |
| Abdominal aneurism, diagnosis of | viii | 464 |
| apoplexy in infants | xv | 248 |
| auscultation, deceptions in | viii | 371 |
| canal, first trace of | i | 389 |
| congestion, leeches to anus in | x | 207 |
| disease in cerebral affections | ix | 475 |
| effusion, case of | xiv | 126 |
| percussion in | i | 487 |
| emphysema, case of | xxi | 470 |
| inflammation, diagnosis of | xii | 280 |
| inflammations, bleeding in | xiv | 5 |
| murmurs, Dr. Hope on | viii | 461 |
| muscles, action of, in labour | xix | 62 |
| percussion too seldom used | i | 480 |
| plethora—a cause of phthisis | i | 77 |
| remarks on | xiv | 330-1 |
| pregnancy, cases of | xxiii | 278 |
| pulsation, Mr. Crisp on | xxiv | 413 |
| rheumatism | vi | 356 |
| tenderness, diagnosis in | xix | 102 |
| remarks on | xxi | 97 |
| tumour, case of, by Mr. Liston | xxii | 375 |
| removal of, fatal | ii | 260 |
| tumours, Dr. Bright on | vi 72, vii | 462 |
| and aneurism | viii | 464 |
| typhus, anatomy of | xv | 96 |
| in Vienna | xix | 366 |
| use of alum in | xix | 366 |
| viscera, ancient ideas of | xv | 446 |
| diseases of | xix | 21, 26 |
| ectopia of, through the diaphragm | xxiv | 179 |
| size and weight of | iii | 199 |
| ABDU-L-LATIFF, biography of | xix | 534 |
| ABERCROMBIE, Dr., account of | xix | 312 |
| Dr. Marx on | xv | 20 |
| enormous brain of | xix | 314 |
| fatal disease of | xix | 314 |
| funeral of | xix | 314 |
| his character | xix | 315 |
| obituary of | xix | 312 |
| on diseases of the brain | iii | 1, 301 |
| remarks on his style | xv | 429 |
| on signs of spinal disease | iii | 30 |
| works of | xix | 313 |
| Aberdeen lunatic asylum | ix | 144 |
| lunatic asylum, report of | vii | 2, 52 |
| statistics of suicide in | iii | 270 |
| ABERNETHY and constitutional irritation | ii | 1 |
| remarks on his practice | xiv | 331 |
| Abkaree, or Bengal bazaar opium | xvi | 355 |
| Abortion, applications of the term | vi | 81 |
| arts of females to procure | ix | 40 |
| cold bathing preventive of | vi | 85 |
| criminal, American law of | iv | 98 |
| English law on | ix | 39 |
| hydatids in | iv | 98 |
| on evidence of | ix | 40 |
| diagnosis in, by Dr. Kohl | i | 110 |
| diseased ovum a cause of | vi | 83 |
| dissent from M. Chailly on | xiv | 367 |
| Dr. Copland on | vi | 81 |
| Dr. Dewees on | vi | 81 |
| Dr. Jörg on | vii | 135 |
| Dr. Lee on | vi | 81 |
| Dr. Ryan on | vi | 81 |
| low estimate of | vi | 81 |
| Dr. von Siebold on | vi | 81 |
| early, like menorrhagia | vi | 84 |
| fuligo splendens, against | xxi | 472 |
| in relation to menstruation | vi | 83-4 |
| in the lower classes | vi | 82-3 |
| medico-legal case of | xii | 562 |
| mistaken for dysmenorrhœa | vi | 84 |
| most frequent cause of | viii 51, x | 87 |
| Mr. Streeter on | x | 548 |
| new forceps for | xx | 526 |
| observations on | xxi | 90 |

Abortion, on bloodletting in vi 85

causes of vi 82

on hemorrhage preceding vi 84

on inducing ix 38

on pains in vi 84

on uterine rigidity causing vi 82

plugging the vagina in vi 85

practical point in x 82

Prof. Dugès on vi 81

progress of vi 84

remarkable case of vi 236, xii 431

sponge as a plug in vi 85

symptoms of vi 83

treatment of iii 408, vi 85, viii 44

two chief symptoms of vi 83

use of ergot in inducing ii 276

use of ergot of rye in vi 86

wilful, criminality of vii 133, 135

Aboussohn, M., on worms in the air-passages ii 249

Abscess, abdominal, case of, by Mr. Liston xxii 375

case of ileo-cæcal viii 279

chronic, of tibia, treatment of xxii 172

formation of, explained xviii 112

hepatic, cured by puncture xv 246

in India xxiii 149

Mr. Geddes on iii 340

rules for exploring xiii 360

intra-pelvic xx 537

in walls of the bladder xiii 237

mammary, compression in xii 259

milk, on opening xxii 157

mistaken for œdema of glottis xiii 230

necessity of rest in vi 156

novel mode of evacuating v 608

of breast, deep-seated xiii 264

of labia, caution on xxi 88

of lungs, pointing distantly xiv 579

of œsophagus, case of fatal xiii 230

pelvic, cases of xxiii 291

penetrating the chest vi 234

perforating the lung iv 317

retro-pharyngeal, case of xx 558

report on xvii 558

secondary, Dr. Nasse on iii 502

symptoms of iii 505

Sir C. Bell on opening vi 156

spinal, case of iii 222

of the liver, Dr. Budd on xxi 46

exploratory puncture of xii 89, 90

mode of opening xxi 48

treatment of xii 89

see Hepatic abscess.

Abscesses and ulcers, hemorrhage from xxii 372

after operations, remarks on ii 10

bad effects of squeezing xi 451

chronic, Sir B. Brodie on opening iii 125

direction of by fascia x 54

hepatic, exploration of xv 246

iliac, after delivery ix 460

in abdomen, remarks on iv 48

Abscesses in iliac fossæ, statistics of ix 458

in neck, mode of opening ii 561

M. Civiale on urinary x 574

M. D'Arcet on multiple xv 131

M. Josse on applications to iii 124

metastatic, theory of xx 218

of the lungs, remarks on xiii 557

on cold water to iii 124

on opening iii 125

on sympathetic ix 458

pelvic, treatise on xix 559

secondary, Mr. Travers on ii 11

subcutaneous, opening of xiii 241

Absence of mind, observations on xv 419

in excess, nature of xxiii 235

Absorbent glands, structure of xiv 290

system of vegetables iv 521

Absorbents, inflammation of, iodine in viii 51

Absorption and reparation, remarks on xxiii 588

and secretion, physiology of xiv 564

and ulceration, on xx 291

by blood-vessels, report on xix 280

by lymphatics questioned viii 312

cutaneous, discussion on viii 313

Dr. Madden on vi 332

experiments on vi 334, 338

from the air vi 338

in the bath vi 334

of gases vi 340

of medicines vi 339

of solids vi 339

of turpentine vi 339

remarks on v 344

Dr. Carson on ii 599

Dr. Jackson on i 156

endosmose in relation to xxiii 390

Hoffman and Magendie on v 346

Hunter's views on xv 207

in animals and plants vii 178

Martigny's experiments on vi 334

medicines promoting xvi 194

Mr. Paget's report on xxi 564

nutritive, Dr. Carpenter on xv 268

of bone, curious case of v 244

of remedies, Dr. Paine on xv 147

on medicines influencing viii 356

phenomena of viii 311

physiology of xix 278

report on xvii 259

retarded by vascular congestion xvi 192

Seguin's experiments on vi 335

selective, and imbibition xiv 564

Abstinence, benefit of in disease v 189

from food for ten days xix 565

system, harvest work on xxiv 536

total, from strong drinks xxiv 523-4

benefits from xvii 53

high medical authority for xxiv 516, 522

in hot climates xxiv 531

medical attention to xxiv 515

on fanatics in xxiv 523

remarks on viii 528

unadvisable ii 388

ABULKASEM, his treatise on midwifery xiv 84
Academicians, on discussions of . xviii 152
Academy, medical, Dr. Gunther on an i 192
of naturalists in Prussia . ii 578
Royal, of Medicine, memoirs of . . . xviii 152, xxi 408
of Science in Russia, founded i 602
Acardiac fœtuses, circulation of . xiv 198
on the circulation in . xvii 491
Acaricide par excellence . . xii 98
Acari, insinuating manners of . xxii 546
of itch, observations on . x 564
Acarus Crossii, observations on . xix 170
folliculorum, discovery of . xv 398
in acne, Mr. Wilson on the . xv 398
of the itch, M. Gras on the . iv 513
scabiei, drugs fatal to . . xii 98
Mr. Wilson on the . xv 397
Accelerator urinæ, on the action of xix 511
Accessorius ganglion, or nucleus . xviii 137
Accessory and first cervical nerve . i 550
nerve of Willis, origin of . xviii 136
report on the . . . xxii 284
nerves, experiments on . . xix 581
Accidents, deaths from, in eight trades i 199
Accomplishments, female, remarks on xi 426
Accoucheur, the, by Mr. Craig . viii 467
Acephalocyst, causes of death from vii 463
Dr. Bright on . . . vii 462
on opening . . . . . vii 464
spontaneous cure of . . vii 463
treatment of . . . vii 464
tumour, diagnosis of . . vii 462
of the bladder . . vii 463
Acephalocystis, account of . . xx 407
endogena, Mr. Owen on . xvii 195
Acephalocysts frequent in hogs . xxiii 69
in man, account of . . xvii 197
inoculation with . . xx 414
in the brain . . . xi 524
or hydatids, remarks on . xvii 194
Acetate of lead and opium in pneumonia xx 81
of lead, experiments with . iv 208
in diseases of the ear . iii 94
in hernia . . . vi 537
on the action of . . iv 208
of morphia in arthritis . . ix 252
Acetic acid in scarlatina . . xxi 497
in hydruria of the horse . xxiv 86
Acetous ether in nervous deafness . iii 99
ACKERMANN, Dr., works and opinions of xix 535
ACHILLINI, Professor, biography of xix 535
Achorion Shönleini, on the . xxiii 509-10
Acid baths, how made . . . i 497
in cutaneous diseases . i 497
carbonic, of the blood, on the i 590
cure, Professor Schultz on the xvi 228
elixir, of Haller . . . vi 329
free sulphuric, detection of . xvi 78
hydrocyanic, detection of . xvi 78
history of . . . xiii 57
muriatic, in the fever of growth ii 220
tannic, preparation of . . xiv 440
Acidity, predisposing cause of disease xx 341
proneness of food to . . xvii 33
Acids, how they aid digestion . iv 203
hydrogen the acidifying principle of xi 140
in the syphilides . . . xxiii 369
mineral, as poisons, tests for . xx 327
principle of affinity in . . xi 146
Acidulous principle of aliment . xvii 9, 14
Acinus, a parent cell with progeny xv 280
Acne, a disease of follicles of skin . ii 150
animalcule discovered in . xv 398
M. Rayer on . . . ii 150
Aconite as an antiphlogistic . . i 249
as a preventive of scarlatina . xvii 223
cautions on the use of . xx 469
death from . . . . xx 466
in diseases of the heart . i 249
its action on the system . xx 465
its effects on animals . . i 249
on the symptoms from . . xx 466
therapeutic action of . . xx 467
tincture of, formula for . . xx 468
use of in inflammations . . xi 327
Aconitine, account of . . xiii 60
Dr. Turnbull on . . ii 499
embrocation . . . xiii 60
ointment . . . . xiii 60
Aconitum napellus, Dr. Fleming on xx 464
in headache . . v 275
Acouophonia, or cophonia, of M. Donné ix 330
Acoustic chair, Mr. Curtis's . v 404
Mr. Curtis's marvellous . xiii 512
instrument improved . . vi 577
Acorn coffee for cachetic children xvii 378
Acorns, use of, as food . . . xvii 16
decoction of, in dysentery . xx 240
Acquired peculiarities hereditary . xiii 167
Acrita, no nervous system found in the v 493
Acrodynia, Professor Romberg on xvii 412
Actinia coriacea, anatomy of the . v 211
Action without power . . . vii 498
Actions, human, nature of all . xx 7
Activity of mind, observations on . xiv 310
ACTON, Mr., on a double monstrosity xxiv 325
on venereal disease x 382, xii 362, 373
Actual cautery, observations on the xxii 172, 397
Acupuncture, cure of neuralgia by . ix 251
in hydrocele and ascites . . v 279
in neuralgia . . . . v 276
use of in ganglions . . xxii 54
ADAMS, Dr., his life of Hunter . iv 75
ADAMS, Mr. F., his Paulus Ægineta, vol. II. . . . . . xxiii 251
his translation of Ægineta . xx 206
ADAMS, Mr. J., advertisement of . xii 518
on amaurosis . . . xii 517
ADAMS, R., on diseases of the joints x 330
Adaptation, general, in nature . xxi 112
Adaptiveness of animals to variation xxiv 67
power of, in matter . . xxiv 188
ADDISON and BRIGHT on the practice of medicine . ix 461, 494
Dr., his practice of medicine . iii 452

Addison, Dr., on fatty liver . . iii 155

on phthisis . . . . . xxiii 421

on pneumonia . iv 356, xviii 337

Addison, Mr., on cerebral and renal disease . . . . . ix 370

on polygastric animalcules . xv 274

on blood-corpuscles, &c. . xvii 92

on nutrition and inflammation xx 121

on the process of nutrition . xviii 91

on the anatomy of the lungs . xiv 59

Adhesion, healing by . . . xviii 280

Sir C. Bell on . . . vi 156

congenital . . . . viii 12, 14

Adhesions, morbid, age of . . xii 134

of peritoneum, diagnosis of . ii 167

Adhesive attraction, Dr. Daubeny on xi 144

inflammation, Mr. Mayo on . iii 61

remarks on . . . iii 62

plaster, Mr. Liston's improved v 459

of Hamburg codex . ii 255

straps, applied to burns . i 580

Adipocire, period of its formation . ii 398

Adipose disease of heart, signs of . viii 464

tissue, Dr. Geddings on . i 153

tumours in small intestine . xxii 474

Sir B. Brodie on . . xxii 171

Adjusting diseases . . . xx 433

Adult stomach, figure of the . ii 540

Adouin, Dr., on the arachnida . i 537

Adventitious products, definition of xxi 422

development of . . xxi 418

structures, Dr. Hodgkin on . xviii 369

Advice, impressive, a father's to his son . . . . . . i 211

giving, results of . . . xxiii 545

Ægineta, Paulus, by Dr. Adams, vol. II xxiii 251

translation of . . xx 206

Ægophony, on the production of . xiv 177

Aeration of the blood, on imperfect vii 264

Æsculapius, his temples in Greece xxiii 90

the Greek temples of . . xvii 457

Ætius of Amida, midwifery works of xiv 83

Affinity, chemical, and capillary attraction . . . . . xxiii 384

Affluence, why favorable to longevity i 196

Affusion, cold, M. Josse's method of iii 115

of cold water in surgery . iii 114

Africa, central, tribes in, account of xxiv 446

health of black troops in . xi 371

Western, bowel complaints in xi 374

hepatic diseases in . xi 374

pulmonary diseases in . xi 374

western coast, humidity of . xi 366

white races of men in . . xxiv 443

African and yellow fever, identity of xi 369

cachexy, description of . . iii 217

coast, climate and diseases of xi 364

fever, Dr. M'William on . xvi 259

Dr. Pritchett on . . xvi 505

in relation to humidity . xi 370

marshes . xi 373

on the cause of . . xvi 506

African fever, treatment of . . xvi 505

languages, remarks on . . xxiv 449

nations, common origin of . xxiv 450

origin of, obscure . . xxiv 480

physical characters of . xxiv 449

survey of . . . xxiv 442

negro tribes . . . . viii 383

station, health of navy on . xv 8

west-coast-fever, nature of . xi 368

After-action of organs . . . xxiv 301

-pains, caution on . . . xxi 91

-pock in the cow . . . ix 95

Agardh, M., quotation from . iv 9

Agaric, cones of, their use for compression . . . . ii 267

Age, advanced, increase of heart in xxii 422

at death, of different classes . xx 422

of 1000 clergymen . i 200

of 1000 medical men . i 200

critical, of women in France . xx 138

of women, remarks on the ii 513

declining, Riverius's periods of xvii 101

influence of, on health and mortality . . . . xxiv 111

in medico-statistical details . iii 270

in relation to diet . . . xvii 42

to phthisis . . . xxiii 438

its effects on the lungs . . i 233

mature, on extending the term of i 324

mean, at death, in 42 professions i 194

of medical practitioners . xxi 444

old, and decrepitude, on . i 323

Dr. Canstatt on diseases of xvii 100

gradual death in . . xvii 44

on the teeth as a test of . v 205

when decline commences . xvii 101

Aged, on the erotic longings of the xvii 84

Agents, medicinal, Liebig on . xiv 516, 519

organic, Dr. Prout's notion on xxi 120

Ages, averages of, correction of . xvii 407

liable to typhoid fever . . ii 52

Agglutination in language . . xxiv 53

Agheustia, in facial paralysis . xxii 282

Agnodice, the instructor of . xv 113

Agony from stroking hair of neck . i 460

Agreement, a common, among physicians necessary . . xxiii 39

Agricultural labourers, salubrity of xxi 10

Agrypnia of old age, remedies for . xvii 110

Ague and fever, distinct nature of . ix 467

and phthisis, antagonism of . xx 217

relation of . . . xx 105

bleeding in cold stage of . ix 468

bloodletting in cold stage of . xvi 563

cases of, treated by salicine . i 571

dead or dumb, treatment of . ix 469

-Dick, a parodied chanson . xv 131

Dr. Berndt on treatment of . ii 176

Dr. Leitao on . . . v 558

effect of civilization on . . xviii 253

quinine in . . . xii 94

endermic use of quinine in . ii 178

Ague, epidemic, of Persia . . xvi 558

Fleischmann's and Skoda's treatment of . . . . . xxiii 607

formula for, by Dr. Berndt . ii 177

hereditary transmission of . xx 211

homœopathic treatment of . xxii 576

homœopathy in . . xxiii 608

Indian, cæsalpinia bonducella in iii 348

use of the chiretta in . iii 348

in early childhood . . xxiii 305

ligature of the limbs in . viii 251

mistaken for typhus . . ix 467

M. Piorry on the spleen in . xii 93

nature of . . . . . xx 347

on bark and arsenic in . . ix 469

on effervescing draughts in . iii 183

on pain of back in . . x 557

pathology of . . . . ix 468

prussiate of iron in . . ii 178

quartan, use of hellebore in . ii 177

salicine unsuccessful in . ii 178

spinal source of . . . ix 468

spontaneous cure of . . v 560

tables of various treatment of ii 176-7

the type of all disease . . xv 125

transmission of to an infant . xii 246

treatment of . . . . ix 468

use of narcotine in . . viii 263

use of piperine in . . ii 178

various medicines for . . xxi 212

with local inflammation . ix 470

Agues in Bengal, complications in . iii 348

Indian remedies for . iii 348

treatment of . . iii 348

venesection in . . iii 348

Indian, the Kutkuleja nut in . iii 348

on bleeding in the cold stage of iii 104-5

on relapse-periods in . . xxi 529

Ahrensen, Dr., on the endermic method v 343

Aikin, Dr., on the fucus esculentus ix 222

Air and exercise, effect of on health xx 109

Air-cells, morbid anatomy of . xi 405

mucous membrane of the . xi 405

of the lungs, formation of the xiv 573

Mr. Addison on . . xvii 94

Air-changes in respiration . . xxi 553

Air, cold, inspiration of in bronchitis xx 449

density of in London before cholera i 350

douche of Eustachian tube, decried xiv 250

in the Eustachian tube iii 95-6

into Eustachian tube . viii 94

Dr. Dunglison on dry and moist i 347

dry cold, most dangerous to life xxiv 113

effects of at great heights . xvii 277

entrance of into veins . . xxiii 443

foul, in mines, remarks on . xv 82

fresh, quantity of necessary . xix 10

how it may enter the veins . vi 462

importance to health of pure open v 406

in hernial tumour, case of . xxii 203

injections of, in ileus . ix 221, 250

in motion, cooling effect of . xix 11

in respiration, quantity used . vi 580

Air in the evolution of the chick . x 229

in veins, cases of, by Dr. Warren i 162

cases of, fatal and not . vi 468

compression and suction for vi 525

cause of death from . vi 465

death from . . vi 457, 460

during operation . . xx 87

experiments on . vi 456, 458, 517

fatal case of . . v 420

history of . . . xxiii 444

introduction of . . vi 455

period of death from . vi 464

post-mortem appearances vi 458, 464

remedies for . . vi 466, 525

signs of . . . . vi 463

symptoms from . vi 457, 463

mechanical properties of . xix 8

memoranda of facts on . . xix 9

mode of excluding in operations xii 251

moist, in baths of Nero, effects of xix 267

of respiration, quantities of xvii 152

on its effect on wounds . iii 127

on introduction of into veins . xii 457

on pure and impure . . xix 2

on various gases in . . xix 13

passage of into the veins, case of xiv 555

passages, on diseases of . xxiv 482

foreign bodies in the v 440, xi 405

on mucous membrane of xxiv 505

plant, observations on the xxiii 493

pump, Sir James Murray on the iii 478

purification of . . . xix 14

purity of, means of testing . xix 5

resident in lungs, volume of . xvii 153

supplementary of lungs, volume of xvii 153

tubes and lungs, contractility of xi 218

auscultation of foreign bodies in v 440

complex affections of the . ix 489

vitiated, a cause of deafness . xviii 501

Dr. Reid on . . . xix 13

Aitkin, Dr., his physiology vii 214, 216

Aix-la-Chapelle, mineral waters of . xiv 346

Akesios, Professor Marx's . . xix 121

Akesius, the healer, account of . xix 121

A-Ke, the Chinese monster . . xii 376

Alæ nasi, defects of, treatment of . xxi 300

Albers, Dr., his pathological observations . . . . iii 220

faults of . . . . xvi 381

his observations in pathology . v 181

on diseases of the cæcum . ix 445

on fatty disease of liver . viii 554

on inhalation of chlorine . iv 212

on pathological anatomy . xvi 381

on putrescence of the uterus . iii 246

on tubercles . . . iv 216

Albinism and albinos, remarks on . viii 20

Albinoism, nature of . . . xxiv 69

Albumen, abnormal, in urine . viii 136

change of, by digestion . . iv 202

into fibrin . . . xv 269

coagulability of . . . viii 132

composition of . . . xvi 283

Albumen, conversion of, Dr. Prout on xvi 484

dysentery treated with . . ix 552

granules in sputa . . xvii 520

manufacture of . . . xix 169

nitric acid best test for . . viii 133

not antidotal of corros. subl. . xiii 574

of blood, Magendie on . . viii 348

of egg and serum, difference of viii 348

injected into veins . . viii 344

on dropsy from deficient . xvii 152

ovi and serum, mixture of . viii 350

properties of . . . . viii 348

reagents affecting . . . viii 349-50

reagents for detecting . . viii 134

the conversion of into fibrin . xv 143

vegetable and animal . . xvi 283

Von Bibra's remarks on . . xxiv 287

and fibrin, difference of viii 348-50, xvi 284

Dumas on . . . xvii 428

M. L'Heritier on . . xvii 428

identity of . . . xii 563

Albuminate of potash . . . viii 348

Albuminous antidote for corrosive sublimate . . . . . xviii 542

fluids, experiments on . . vi 74

impregnation, cause of . . x 325

tissues, metamorphosis of . xv 506

urine, diseases with . . xiii 262

disquisition on . . ii 224

division of . . . xi 343

from renal congestion . xvii 494

Dr. Barlow on . . x 301, 327

Dr. Graves on . . xvi 243

in some dropsies . . vii 163

M. Piorry on . . vi 142

Albuminuria after scarlatina, cause of xvii 484

causes of . . . . . xx 265

crisis indicated by . . viii 142-4, 157

diagnosis of . . . . viii 249

Dr. Prout on . . . xi 344

from alkalies . . . viii 140

from cantharides . . . viii 140

from essential hæmaturia . viii 139

from mercury . . . viii 140

from morbid blood . . viii 138

from semen . . . viii 141

in acute diseases . . . viii 142-3

in diabetes . . . . viii 139

in dropsy . . . . viii 145-6

in healthy persons . . . xiii 444

in several diseases . . . xiii 444

in various diseases . . viii 146

M. Solon on . . . x 301, 328

observations on . . . xiv 578

on the causes of . . . xx 107

or dropsy, M. Solon on . viii 121

pathology and treatment of . xx 266

propositions respecting . . viii 147

table of causes of . . viii 137

temporary . . . . viii 139

treatment of . . . xxiv 44

urine and blood in . . xxiv 45

Alcmæon, anatomical improvements of xv 106

Alcock, Dr., on the fifth nerve . iii 257

on the nerves of taste . . iii 259

Alcock, Mr., on injuries of the joints xi 459

on the Spanish legion . . vi 448

rare operation by . . vi 452

Alcohol and brain, affinity between viii 540-1

and olefiant gas, relation of . xi 134

absorption of, by the stomach xxiv 544

action of, on the blood . xvi 218

on spleen and liver . xvi 219

bad effects of habitual use of . xxiv 528

cannot add to muscular vigour xxiv 525

consumed in Britain and Ireland . . . . xviii 116

consumption of in America . xviii 116

effects of, on man and animals xiii 159

heat-producing powers of . xxiv 527

in French drinks, quantity of xviii 116

in medicine, on the use of . xxiv 541

in morbid exhaustion . . xxiv 544

in relation to digestion . . xxiv 529

insidious support from . . xxiv 547

in the brain, inquiry on . . viii 539

its effects on the blood xxiv 526-7, 531

mode of obtaining from organs viii 540

on its action on the system . xiv 503

on its utility in fever . . xvii 355

on the modus operandi of . viii 540

on the production of heat by . xiv 503

relation of to nervous system xxiv 526

use of, in warm climates . xxiv 530

weakens in hot situations . xxiv 528

Alcoholic drinks, &c. . . xvii 22

Dr. J. D. Hooker on . xxiv 532, 534

physiological effects of . xxiv 515, 525

fluids in fever, remarks on . xvi 361

liquors as stomachics . . xxiv 543-4

reputation of as tonics . xxiv 547

use of as stimulants . xxiv 545

lotion for phthisis, Dr. Hall's . xxiii 187

principle of aliment . . xvii 10, 14

stimulants in dyspepsia . xxiv 546

Alcyonella stagnorum, paper on the v 212

Alderson, Dr., on diseases of the stomach . . . . . xxiv 273

Dr., on the effects of lead . ix 435

Aldis, Dr., his introduction to hospital practice . . i 490

Aldridge, Dr., his case of rheumatism . . . . ix 275

Ales, strong, qualities of . . xvii 22

Alessandrini, on development of muscle . . . . . ii 543

Alexander, Dr. F. S., on anatomical pathology . xvi 381, 386

on glanders in man . . ii 241

on puerperal fever . . . ii 490

remarks on his style, &c. . ii 492

Alexandria and Cairo, Sir W. Pym on xxiv 244

plague in, 1834-5 . . . i 248

Alexandrian school of medicine . xxiii 93

Alexis, the clairvoyant, in England xix 457, 469

Alfourous, or Alforian races . xxiv 470-1

Algæ, spores of, remarkable motions of xxiv 224
used as food, constituents of xvii 21
Algeria, rarity of phthisis in . xiv 45
Algide intermittent fever . . viii 62, 66
Algiers, the fevers of . . xiv 43
ALIBERT, M. his treatment of tinea i 576
on the treatment of porrigo . i 578
Aliment, insufficient, indigestion from . . . . . xvii 348
Aliments, compound . . xvii 15, 28
conversion of, Dr. Prout on . xvi 480
liquid, or drinks . . . xvii 21
Alimentary canal, action of salts in xi 442
chemical actions in . xxiii 379
primary principles . . xi 330
principle, what is an ? . . xvii 12
principles, conversion of. xi 330, 332
principles . . . . . xvii 7
modification of . . xi 330
Alimentation, insufficient, effects of xvii 347
ALISON, Dr. W. P., sketch of . . iii 193
his outlines of physiology viii 241, xiii 468
his paper on sympathy . . v 533
his practice of medicine xv 533, xviii 213
on epidemic fever . . xviii 179, 182
on inflammation . . . x 235
on instinct . . . . . xi 90
on the blood, in asphyxia . ii 424
on the cause of fever . . xi 23
on the condition of the poor . . . xviii 492, 512
on the poor in Scotland ix 544, xi 1, 23, 36
on vision . . . . . x 1
ALISON, Dr. S. S., on alterations of the heart . . . . xxii 234
ALISON, Mr., on beauty of countenance xviii 445
on population . xiv 446, 450, 461
Alizarine, not changed by ammonia v 175
Alkalies azotized, mostly poisonous xiv 308
intolerance of . . . xi 354
mode of action of, on urine . xxii 224
on the use of, in stone . xii 390
pure, use of, in consumption xiv 196
vegetable, action of . . xiv 308
Alkaline bath, composition of . i 497
in cutaneous diseases . i 497
chlorides, conversion of calomel by . . . . . . xviii 545
mineral waters, M. Petit on . xiv 350
waters . . . . . . xiv 326
Alkaloids, conversion of, into brain, &c. xiv 518
ALKENANI, biography of . . xix 534
Allantoin, chemical formation of . xi 436
Allantois, formation of the . . ix 20-1
in the human ovum . . i 240
of different animals . . ix 21
origin of, in the rabbit . xvi 554
in man . . . . . xvi 556
ALLEN, Dr. A. S., his case of wounds of fingers . . . . iv 25
ALLEN, Dr. M., on the insane . vii 1, 46
ALLEN, Mr. O., on the York dispensary xx 204
ALLNATT, Dr., on tic douloureux . . . xii 524, xvi 511
Allopathy and homœopathy, by the editor . . . . xxi 502
momentous remarks on . xxi 256
Almanac, American medical . xii 223
British medical, notice of . i 216
Farr's British medical . iii 206
medical, Mr. Farr's . . vii 294
Almonds, amygdalon and emulsion in xxi 274
bitter, poisoning by oil of . xviii 560
Aloes, endermic use of . . v 352
Kurachee and Deckan . . xvi 358
on the operation of . . x 447
on the use and abuse of . iii 264
preparations of . . . xii 222
socotorine . . . . xvi 358
Alps, goitre in regions of the . viii 118
Alterations, pathological . xxiii 55, 51
Alteratives, medicinal action of . xii 89
Alum, calcined, in strictures of urethra iii 242
formula of, for hooping cough xxiii 424
injurious effects of in bread . xvii 25
in inflamed mucous membranes xi 524
Amauroses, the elements of the . xvii 169
transformations of the . . xvii 169
Amaurosis and mydriasis, different v 43
asthenic, and cases of . . xvii 156
case of . . . . . v 42
cases of . . . . . iii 518
cause of failure in . . . xvii 155
cause of, in squinting eye . v 42
changes of structure in . . v 45
complete, cured by mercury . v 44
congestive . . . . xvii 158
convex spectacles in . . xv 246
cured by cholera . . . xviii 414
erethistic, and cases of . xvii 159-62
feigned, case of . . . v 45
Mr. Tyrrell on . . v 45
from cerebral disease . . v 44
from lead poisoning . . x 454
treatment of . . ix 435
from lightning . . . xvii 157
from undue lactation . . xii 328
from worms . . . xvii 157
functional, of retina . . v 42
glaucomatosa . . . xvi 115
hyperæmic irritative . . xvii 159
imperfect, treatment of . . vi 74
incautious depletion in . . xviii 415
interesting case of . vii 282, xviii 414
mode of leeching in . . xvii 159
M. Pétrequin on . . . xvii 155
M. Pétrequin's classification of xvii 168
Mr. Hocken's treatise on . x 246
Mr. Tyrrell on . . . v 31, 40
nervous irritative . . xvii 159
new operation for . . xii 517
opalescent . . . . xvi 116
organic, and cases of . . xvii 166

Amaurosis, pathology of . . v 40

relapses in . . . . . xvii 169

remarkable case of, cured . v 44

remarks on the term . . v 40

sanguineous . . . xvii 159

strychnine in . . . . v 42

suggestion respecting . . iv 481

torpid, alleged cases of . . xvii 164

or paralytic . . xvii 163

traumatic, and cases of . . xvii 157

treatment of . . . v 42

use of strychnia in . . xvii 170

varieties of classed . . v 41

Amaurotic cat's eye . . . x 38

myopia, M. Pétrequin on . xvii 169

Ambodik, Prof., his writings, in Russia i 606

Amelot sewer in Paris, account of the v 450

Amenorrhœa, causes and cure of . x 75

Dr. Ashwell on . . . xi 178

dry cupping the breasts in . iii 481

from imperforate cervix uteri . xvii 542

hymen . . . xxiii 291

vagina . . . xxiii 291

from imperforation . . vi 91

mode of using electricity in . xii 345

observations on . . . xiv 392

on electricity in . . . xii 345

on forcing medicines in . . xiv 392

on mercury in . . . iii 399

report on . . . . . xx 539

singular remedy in . . vii 234

Amentia, curious case of, by Dr. Rush xxiii 234

interesting case of . . xx 25

America, the aboriginal people of . xxiv 473

and her prospects . . xiv 454

British diseases of lungs in . viii 222

congestive fever of . . xx 236

fanaticism or ultraism in . v 409

medical institutions in . iii 273

Mr. Combe's notes on . xv 52

North, the climate of . . xiii 423

petty satirical attacks on . xv 59

poisonous cattle in . . xviii 555

South, great salubrity of . xv 6

strictures on education in . v 413

American amusements, account of . v 413

army, barracks of the . xvi 37

mortality in the . . xvi 37

Cyclopædia of Medicine and Surgery i 152

delegates, mean age of at death i 344

Indians, intellectual defect of . x 476

Mr. Combe on . . xv 58

Journal of Pharmacy . . ii 233

journals, medical . . ii 233

reflections on the . . iii 194

languages, structure of . . xxiv 474

lectures . . . . . iv 194

medical almanac . . . xii 223

journals . . . . i 228

library . . iv 495, viii 539

nations, Asiatic origin of . xxiv 476

moral and social state of xxiv 475

origin of syphilis, a fable . xxiii 347

American parson, anecdote of an . iv 57

precocity of refinement . v 410

Pharmacopœia (1842) . xiv 437-41

prison system, insanity from . xii 501

races, division of . . . x 476

truss for hernia . . . v 300

Americans, dress of the fair . . v 412

liberal English feelings to . xv 59

Amesbury, Mr., his apparatus for fractures . . . vi 311, 318

on spinal deformity, &c. . xii 177-8

Ammon, Dr. von, on diseases of the eye x 2, 20

on iritis . . . . x 2, 34

on ophthalmology . . . iv 480

on plastic surgery . . . xix 396

on strabismus . . xi 474, 492

on tenotomy . . . v 550

Ammonia, carbonate of, Dr. Philip, on ii 146

in blood . . . xxii 329

in scarlatina . . xvii 216, 224

detection of, in the fluids . xvi 150

generation of, in the system . xvi 150

hydrochlorate of, dose of . xxiv 504

in air . . . . . . xix 14

injection of, in chlorosis . xi 178

its use in erysipelas . . iii 149

mixture of, for scarlatina . xvii 224

muriate of, use of . . . xix 241

natural source of . . . xi 440-1

use of, as a counter-irritant . vii 68

Ammoniacal salts, effect of, on blood xvi 153

Ammoniated copper in diabetes . ii 180

Amoros, Colonel, his gymnastics . xx 109

Amnion, development of . . ix 20

origin of, in man . . . xvi 555

in the rabbit . . . xvi 550

Amphibia, Mr. Macilwain on . vi 101

urinary organs of . . . xii 132

Amphoric resonance in chest, cause of vii 570

remarks on . . . i 483

Amputation, a new method of . viii 205

arterial compression in . . xiii 172

at the hip-joint . . . xiii 173

case of . . . xxii 113

operation of . . . xxii 111

at the knee-joint . . . xx 55

burying nerves in . . . vi 168

causes of fatality of . . vi 563

dressing after . . . vi 168

figures of oblique . . . viii 206-7

flap, history of . . . xvi 64

operation of . . . xiv 61

Sir C. Bell against . . vi 167

history of . . . . . xvi 63

in Africa, statistics of . . xii 260

merits of flap and circular . xv 191

Mr. Alanson's mode of . xvi 65

Mr. B. Cooper on . . . ix 392

Mr. Guthrie's precept on . vi 452

Mr. King on . . . . xiii 172

Mr. Liston on . . . xxii 376

in compound fractures . . xiii 172

in gangrene, M. Josse on . iii 123

Amputation, Mr. Travers on . . ii 28

issues before . . . . vi 563

oblique, method of . . viii 206-9

section in . . . viii 205

of cervix uteri, Duparcque on ii 517

of child's arm in labour . ii 526

of limbs, observations on . xxii 52

of foot . . . . . vi 535

of leg, place for . . . xiii 565

of legs, cases of double . . vi 550

of limbs in utero . . iv 467

of penis, Dr. Môrrison on . iii 266

of upper maxillary bone . xx 195

on delay of . . . . xx 54

on Dupuytren's mode of . vi 168

on primary or secondary . vi 452

on the results of . . . vi 561

position of surgeon in . . xx 44

Professor Chelius on . . ii 259

rules for . . . viii 210

secondary, Mr. Fergusson on . xxi 484

Sir C. Bell on . . . vi 166

spontaneous, in fœtus . . ix 564

in utero . . v 614, viii 567

statistics of . . . . . xi 256

treatment after . . . vi 562

when well performed . . vi 167

Amputations, circular and flap . vi 167

of Dupuytren, fault of . . xiii 173

purulent deposits from . . xi 462

statistical account of . . xiv 60

results of . . . . iii 237

use of heat in . . . . xix 187

Amputating apparatus, caution on . xiii 172

knife, plate of new . . . viii 206

Amsterdam college and medical school vi 277

Society, transactions of . xiii 503

Amulets, Turkish, account of . iv 382

Amusements of the rich and poor xiv 310, 311

Amussat, M., his operation for stone xii 410

on air in the veins . . vi 456, 517

on artificial anus . ix 259, xviii 452

on stammering . . . xii 212

self-contradiction of . . xviii 458

Amygdalin, on the medical use of . xxi 274

Amylaceous principle of aliment . xvii 9

substances, digestion of . xxiii 393

Anableps, singular eye of the . . vii 228

Anæmotrophy different from anæmia xi 346

Anæsthesia, cases of . . . xxiii 413

from lead . . . . . x 453

in etherization, remarks on . xxiii 555

in operations, by ether . xxiii 309

from ether . . . xxiii 547

of fifth nerve, case of . . vii 544

Analogy between instinctive, reflex, and emotional actions . xxiv 207

of brain, spinal cord, and sympathetic ganglia . . xxiv 203

on arguing from, in science . xiv 78

and homology, on the terms . xxiii 475

Analogism of the empirics . . xxiii 101

Analysis, chemical, Dr. Fresenius on xvii 244

Analysis, chemical, improvement in . xi 134

Analytical processes in the system xvi 150

Anamnesis in diagnosis . . xx 309, 315

Anarcotina, (narcotina), properties of xvi 356

Anasarca and fits after scarlatina . ix 276

after scarlatina . . . xvii 221

kidneys in . . iii 435

treatment . . iii 436

danger of scarifications in . iii 432

diuretic, formula for . . iii 431

Dr. Seymour on . . . iii 430

Dr. Seymour's theory of . iii 434

fatal symptom in . . iii 433

following scarlatina . . iii 435

from chlorosis and loss of blood iii 436

from cold bathing . . . xxi 369

from disease of the heart . iii 430

of the kidneys . . iii 436

from enlarged heart . . iii 431

and thin heart . . iii 432

on the use of cantharides in . iii 432

relieved by acupuncture . iii 432

supertartrate of potash in iii 432, 436

treatment of . . . iii 431-2

use of digitalis in . . . iii 432

of elaterium in . . iii 432

with albuminous urine, case of iii 435

without organic disease . . iii 435

Anastomosis in ligature of the aorta xxii 108

Anatomy, abuse of, by artists . xiv 528

and histology, special . . xviii 223

and physiology, cyclopædia of i 536

Dr. Budd on . . xx 509

Mr. Walker on . . xiii 205

report on . xxi 541, xxii 261

and therapeutics, relation of . xiv 197

comparative, aided by geology i 392

cultivated by Aristotle . i 380

Dr. Grant on . . xiii 217

general fact in . . xxii 497

in studying the brain . xxii 489

its importance . . i 537

Mr. Jones on . . xii 218

remarks on . . . i 393

use of . . . . xviii 423

utility of . . . viii 506

Crommelinck's . . . xiii 519

demonstrations of . . xi 220

descriptive, study of . . x 190

Dr. Krause's manual of . . vi 204

Dr. Lizars' elements of . . xix 243

Dr. Quain's elements of . v 216

Dr. Weston's system of . viii 244

elements of . . . . . xxi 499

first steps to . . . . xxi 493

general, initiated by Bonn . i 388

manual of . . . iv 497

treatises on . . . xiv 478

manual of, by Dr. Krause . iii 198

microscopic, Mr. Hassall's . xxii 552

Mr. Hassall on . . xxiii 252

morbid, Dr. Carswell's . . i 117

Dr. Carswell's plates of . i 152

Anatomy, morbid, Dr. Hodgkin's iv 31, 55, xi 401

four species of i 118

French school of xi 400

illustrations of i 117

its use in nosology i 28

Mr. Travers's remarks on ii 30

obligations of medicine to xi 400

on the neglect of xxiii 65

remarks on vii 116

Sir A. Carlisle on vi 189

treatises on xv 83

M. Broc on teaching xiii 519

Mr. Paget's report on xvii 249

Mr. Wilson's surgical viii 239

new edition of Soemmering's xiv 373

of breast, Sir A. Cooper on x 101

of expression, Sir C. Bell on xviii 442

of structure, history of xiv 482

of the ancient Hindoos xxiii 537

of the regions, &c., Dr. Lebaudy's ii 196

on education in x 180

pathological, arrangement of iv 31

essay on xix 362

Dr. Gross's xxiii 64

Dr. Vogel's xxii 323, xxiii 250

its value i 400

remarks on xiii 328, xviii 48

treatises on xvi 381

philosophical science of vi 107

physiological, of man xv 537

on the study of x 180

pictorial, lecture on xiv 528

report on progress of xix 249, 563

surgical, history of x 347

scope of x 347

treatises on x 347

teachers of, regulations on iii 292

regulations concerning ii 610

text books on ix 538

transcendental, remarks on viii 484

textural, Prof. Burggraeve on xxiii 136

use of nervous comparative xiii 480

utility of, to artists xviii 442

von Behr's handbook of xxiii 255

Anatomical characters, mistake on xii 98

dissection, remarks on viii 240

dissections, ancient xxiii 95

drawings vii 224

education, remark on xxi 494

language, lax, ancient xv 107

observations, Mr. Goodsir's xx 289

period of medicine xxiii 93

plates, Hindustani xxii 227

Langenbeck's xiv 539

researches, Dr. Davy's xii 129

sketches and diagrams viii 542, xii 521

by Wormald, &c. vi 202

specimens, new casts for vii 278

subjects, preservative of ix 397

Anatomico-pathological form of disease xxiii 43, 54

Anatomist's Vade-mecum x 547, xiv 220, xix 243

Anazoturia, nature and treatment of vii 159

Ancell, Mr., singular case of tumours by xv 153

Anchylosed knee, sudden force in xiii 14-20, 28

Anchylosis, agency of muscles in xviii 355

diagnosis of xviii 356

Dr. Little's tenotomy in xviii 360

Duval's tenotomy in xviii 360

external and internal xiii 169

exciting cause of xviii 354

extension after tenotomy in xviii 361

false, of knee, cure of viii 400

forcible extension in xii 552

in extremities of a fœtus v 579

internal, causes of xiii 169

M. Lisfranc on xv 51

Mr. Mayo on the cause of xiii 170

new treatment of vi 254

never from rest or disuse? xviii 354

of hip, artificial joint in xx 50

of hip-joint, operation for x 282

sawing neck of the bone in xiii 170

of joints, on producing x 336

of knee, dissection of xiii 13

false, cure of xiii 13

operation for xiii 12

on the rapid cure of xx 464

osseous, rarity of xiii 170

prognosis of xviii 357

sacro-iliac and pubic xiv 535

state of muscles in xviii 355

temporo-maxillary xiii 527

tenotomy first used for xviii 354

treatment after tenotomy in xviii 362

treatises on xviii 353

treatment of xviii 358

without tenotomy xviii 363

when tenotomy inadmissible in xviii 359

why flexion of the joint in xviii 355

Ancient arts, recent discoveries of xxiii 521

medical biography xv 106

writers, the study of xvii 470

world, Professor Ansted on the xxiv 281

Ancients, on the physiology of the xv 442-52

Acon breed of sheep, in America vii 376

Anderson, Dr., treatment of lunatics xvii 285

Anderson, Dr. A., on typhus fever xii 294, 317

Anderson, Mr., his anatomy of the nervous system iii 491

Andral, M., his microscopic defects xvii 137

his notes on Laennec v 423

his mistake on fibrin xvii 138

on diseases of the chest iv 285

on the pathology of the blood xvii 136

on the state of the blood xi 242

on his education iv 286

Andral's, Clinique Médicale, translation of i 188

Andreæ, Dr., on ophthalmic medicine x 1, 20

Anæmia, arterial murmurs in viii 459

murmur in xvii 141

cases of, in dressmakers xxiv 44

Anæmia, diagnosis in . . . xxi 89

determinations of blood in . xvii 487

encephalic congestions in . xvii 488

general, Dr. Williams on . xvii 486

M. Andral on . . . xvii 140

on nervous excitement in . xvii 486

or oligemia, Dr. Canstatt on . xiii 332

treatment of . . . x 67

spontaneous, the blood in . xvii 140

treatment of . . . xxiv 44

Anemic deafness removed by stooping xvii 140

insomnia, cold foot-baths for xxii 54

Anencephali, separate existence of viii 32

Anencephalous monster, how produced viii 16

Anesthesiæ of the nerves . . xvii 419

Aneurism, bellows-sound in . . i 456

abdominal, diagnosis of . . viii 464

and dilatation distinct . . xii 443

aortic-abdominal, diagnosis of xxiv 417

Brasdor's operation for viii 417, xxi 288

British achievements in . . xii 439

authors on . . . xii 439

by anastomosis . . . xxiv 421

coagulation of blood in . . xii 440

cerebral . . . . xxiv 418

collection of treatises on . xx 206

compression in . . . xx 434

advantages of . . xx 438

objections to . . xx 439

crooked and stiff limb in . xii 445

cure of by pressure . . xix 525

definition of . . . vii 149

dissecting, Mr. Crisp on . xxiv 418

of aorta, case of . . xx 94

Dr. Blasius on . . . viii 413

Dr. Wardrop on . . . viii 413

Dr. Wardrop's operation for viii 417-18, xii 448

enormous ventral . . vii 154

false consecutive, of the heart ii 338

from wound, treatment of xiv 27

Lisfranc on . . . xiv 27

Mr. Liston on . . xxiv 326

pathology of . . xiv 27

or dilatation, of the heart ii 338

fibrinous concretion in . . viii 415

fibrous tumour mistaken for . xvi 384

galvanism by needles in . xxiii 407

hemorrhage after operation . xiv 25

hunger cure of . . . viii 416

Hunterian operation for . viii 417

in relation to age, table of . vi 49

to sex and age . . xxiv 415

John Hunter on . . . v 53

lateral circulation in . . xxii 109

mistaken for laryngitis . . xii 446

for pneumonia . . xi 516

Mr. Hodgson on . . . viii 417

Mr. Porter on . . . xii 438

Mr. Thurnam on . . . xi 463

new treatment of . . xiii 564

odd remarks of Mr. Porter on xii 447

of the abdominal aorta . . xxiv 417

Aneurism of the ascending aorta . ix 548

case of . . . . xv 148

of the carotid, case of . . v 610

of external iliac, case of . xx 96

of heart, biloculate . . vii 151

external mixed . . vii 151

forms of . . . vii 150

multiloculate . . vii 151

nature of . . . vii 150

on circumscribed . . ii 460

on death from . . vii 151

symptoms of, obscure . ii 331

of the innominata . . xxiv 420

of mesenteric arteries . . xiv 64

of the orbit, case of . . v 36

of popliteal artery, case of . xxiii 410

of the subclavian . . . xxiv 420

of the thoracic duct . . ii 535

on a somewhat peculiar . xxiv 323

on a variety of false . . xv 155

on diffused and traumatic . xii 448

on medical treatment in . xiv 26

on the causes of . . . xxiv 414

on the nomenclature of . xii 444

on the tunics involved in . xii 444

on two ligatures in . . xiv 24

operation for, Lisfranc on . xiv 24-25

operations for, history of . xvi 62

osseous, fatal case of . . xiv 581

popliteal, case of . iv 256, vii 153

cured by pressure . . ix 277

relapsed, case of . . vi 67

remarkable cases of . . xxiii 416

secondary hemorrhage in . xxiv 419

Sir C. Bell and Scarpa on . vi 169

spontaneous cure of . viii 414, xii 446

subclavian, case of cure of . xii 446

the external incision for . xiv 22

thoracic, cases of . . viii 464

on diagnosis of . . iii 551

pathological effects of . xxiv 416

treatment in plurality of . xii 445

of by ice . . . v 568

of by pressure . xxi 490, xxii 373

tying femoral artery in . xiv 132

varicose, remarkable case of . iv 138

operation for . . xxi 470

varieties of . . . . xii 443

Aneurisms, cause of agony in some iii 25

of the heart . . viii 149, xx 224

Angeioleucitis, chronic . . xiii 178

diagnosis of . . . . xiii 176

from phlebitis . . ii 557

effects of . . . . xiii 177

general symptoms of . . ii 557

local symptoms of . . ii 556

morbid anatomy of . . ii 558

M. Velpeau on . ii 556, xiii 173, 182

symptoms of . . . xiii 174

terminations and prognosis of ii 557

terminations of . . . xiii 174

treatment of . ii 557, xiii 177

Anger, bile in stomach from . . vi 178

ANGELO, Michael, head of . . ix 208
Angels, communication with, case of xix 471
Angina, use of powdered alum in . xiv 228
membranous, contagiousness of xii 537
tonsillaris, treatment of . xxiv 138
Angina pectoris, a neuralgia . . xvii 415
aortitis a cause of . . v 273
cases of . . . xix 25
causes of . . . xvii 416
cured by iodine . . v 165
Dr. Latham on . . xxiii 183
fatal case of . . xi 238
inhalation of ether in . xvii 416
on the nature of . . vi 146
pathology of . . xvii 415
remark on . . . vi 374
treatment of xvii 416, xxiii 184, xxiv 41
use of sulphur in . x 586
women little subject to . xvii 416
Angionosi, Dr. Lanza on . . xxiii 56
Angioitis of the Italian physicians . ix 223
supposed cases of . . ix 224
Anglo-Indian army in Egypt, health of vi 151-4
Angustura bark, false, account of . xvi 358
Anima of Stahl . . . . . i 385
Animalcule of the skin, Mr. Wilson on xxi 200
infusorial, nutrition of . . iv 12
parasitic, cause of contagion . ix 399
Animal and vegetable kingdoms . vii 171
varieties, development of . xix 179
body, Reil's idea of the . . v 79
bodies, mechanical machines of xxiv 194
cells, growth of . . . ix 505
chemistry, Dr. Liebig on xiv 492, 521
Simon's treatise on . . xx 206
treatises on . . . xvii 424
economy, John Hunter on the vii 523
fluids, saline constituents of . xxi 543
food, condemnation of . xxi 153-8
Dr. Lambe against . . v 410
on its quick digestion . ii 542
vomiting from the least . xvii 415
foods, constituents of . . xvii 18
observations on . . xvii 18
forms, extreme minuteness of . xi 137
functions, pathology of the . iii 19
heat, authors on theory of . xiv 297
Chossat's experiment on . xvii 431
Dr. Carpenter on . . xiii 484
Dr. Davy's experiments on xii 139
experiments on . . xiii 229
first truly explained . i 383
Mr. Jeffreys on . xvii 153
Mr. Paget's report on . . xxi 557
not kept up by alcohol . xxiv 526
observations on . . xi 393
on the generation of . xvii 430
present knowledge of . xix 266
irritant poisons, report on . xviii 555
kingdom, affinities of the . vii 171
Dr. J. C. Hall on the . . xiv 218
life, functions of . . . xiii 476
kingdom of . . . xi 102
Animal life, organs of, report on . xvii 261
magnetisers, credulity of . xix 478
magnetism, Academy of Medicine on . . . iv 442, 444
acts at a distance . . vii 327
ancient history of . . vii 338
Andral's belief in . . iv 441
argument in favour of . iv 446
Cuvier favorable to . . iv 441
Dr. Sigmond on . . v 598
of Dr. Elliotson . . vii 348
effect of on a materialist . iv 447
effects of . . . vii 303
Gmelin's views of . . vii 318
history of . . . vii 303
imagination in . . xix 475
in America, 1834 . . iv 445
in France . . . vii 309
in England . . . vii 347
in Germany . . . vii 315
Laplace on . . . vi 196
morality of . . vii 319, 335
Mr. Colquhoun on . iii 1, iv 441
nature of . . . vii 324
procès verbal on, 1828 . iv 445
reflections on . . vii, 304, 352
remark on . . . iii 328
rejected in 1784 . . iv 441
treatises on . . vii 301, xix 428
matters, carbonization of xiii 186, 194
nature as a whole, Unzer on . xxiv 209
organisms, essential elements of xi 334
incidental elements of . xi 334
physiology, Dr. Carpenter's . xvii 241
Dr. Unzer on . . . xxiv 181
soul-force of the brain . . xxiv 186
Animal's friend . . . . viii 536
Animals, capacities for variation in . xxiv 58
captive, consumption in . . xx 104
carnivorous, nutrition in . xiv 504, 515
difference of pain in . . viii 265
diseases of in Berlin, 1837 . vi 220
diseases transmissible from . xiii 28
domestic diseases of . . xxiv 82
epidemic cholera affecting . iii 256
experiments on living . . viii 536
herbivorous, nutrition in xiv 507, 515
infusory, production of . . i 392
instinctive actions of lower . xi 91
knowledge of time in . . xi 92
lower, intellectual powers of xi 92
lowest, motions of the . . v 489
microscopical structure of . ix 495
moral feelings of . . . xi 96
on general notions of . . xi 92
on painful experiments on x 185, xvii 344
on varieties in . . . iii 376
permanent characters of, functional . . . . iii 372
the lower, on dispersion of . iii 369
the lowest, sensibility of . v 489
treatises of the food of . . xxiii 157
Animals, varieties of from climate . xv 181

Animals and man, diseases of compared xxiv 82

and plants, analogy of . . ix 495

analogies in . . . vii 170

development of . . xix 165, 174

dispersion of . . iii 367, 370

origin of . . . iii 367, 370

similar growth of . ix 495, 520

and vegetables, analogies of . . iv 8

distinction of . . iv 6, xxiii 492

Animated nature, Macleay system of xix 181

tribes, origin of . . . xix 168

Ankle, dislocation and fracture of . xiv 41

Ankyloblepharon, treatment of . xxi 309

Annals, new, of medical science . i 219

of medical science (Leipzig) . iii 462

of medicine, universal (Italian) i 223

Annoyance juries, account of . xv 330

Annales de Hygiène, &c. . . i 226

Annesley, Mr., on diseases of India xiii 347

Annuities, national loss from . iii 55

remarks on . . . . . iii 55

Annus magnus of the ancients . xviii 173

Anomalies, congenital . . . viii 18

in the form of organs . . viii 19

numerical, of muscles . . viii 23

various of organs . . viii 22

Anomalous cases, source of many . xii 105

diseases, hydropathy in . . xxii 445

Ansted, Professor, his geology . xix 554

on the ancient world . . xxiv 281

Anstruther, typhus fever at . xii 316

Antagonism, Dr. Henle on . ix 407

of disease . . . . . xx 217

of diseases, M. Boudin on . xxii 212

of visceral nerves . . . xxiv 311

Anteversion of the uterus . . iii 404

case of . . . . . xxiii 292

Lisfranc on . . . xviii 25

Anthelmintics, Abyssinian . . xii 250

Anthony, Dr., in Russia, account of i 600

Anthology, medical, Italian . i 222

Anthrax, epizoot. contagious to man xxiv 86

essential, of Dr. Levin . . xiii 41

malignant, history of . . xiii 43

morbid anatomy of . xiii 43

rapid death from . . xiii 43

or benign carbuncle . . x 350

Anthropology, general remarks on xviii 387

Anti-animal system of living . . v 409

Anticipations of the future, sensational xxiv 191

Antidotes, observations on . . xvi 195

Antidynous lotion . . . . vi 501

Antilithics, Dr. Paris on . . xvi 196

Antimonial and arsenical stains . xvi 76

stain, Mr. Marsh on . . xiv 123

vapours, poisoning by . x 580

Antimony, as an emetic . . xv 72

in emetic tartar, detecting . xvi 77

in the urine . . . . xii 249

its effect on platina . . ix 58

on hydrated sulphuret of . viii 556

on poisoning by . . . xi 52

Antimony, stains of, characters of . xi 40

tartarized, absorption of . xi 52

poisoning by . . xviii 552

preparation of. . . xiii 60

tartrate of, its effects in disease i 415

in head affections . . ii 146

testing for, remarks on . . xvi 77

Antiphlogistic, general, a risk from xvii 483

measures, caution on . . v 387

medication, remarks on . . xxi 161

Anti-phrenologists, the great error of ix 204

Antiquaille, lunatic hospital of . ix 178

Antiseptics, Dr. Paris on . . xvi 196

Antispasmodics, observations on . xvi 192

with purgatives . . xvi 200

Antoninus, plague in the time of v 193

Ants, eggs of, emetic . . . xv 73

Anuria apyretica, cases of . . ix 573

or ischuria renalis . . vii 160

seu urodialysis senum . . xvii 117

Anus, artificial, actual cautery for . ii 567

Callisen's operation for modified . . xviii 462-3

Dieffenbach on the cure of xxiii 31-6

different operations for xviii 453-71

Dr. Gross on . . . xxiii 27

Dr. Riecke on . . xiv 364

fecal fistulæ in . . xii 460

formed in abdomen . . xviii 459

in lumbar region . xviii 463-71

made in groin . . viii 256

M. Lisfranc on . . . xv 40

M. Littré on . . xviii 459

M. Velpeau on . . xii 459

Mr. Trant's instrument for xxiii 27

new mode of forming ix 259, xiii 237

operation for . . xiv 231, 250-1, xxiii 453

operations for, table of . xviii 461

plastic treatment of . vii 415

treatment of . . iii 520

various treatises on . xviii 452

excoriation round, from mercury v 23

fissure of, in infants, cases of xxiii 305

use of rhatany in . . xi 251

history of fissure of the . . v 484

imperforate, case of . xiii 508, xxiii 301

two classes of . . xviii 453

the sphincter in . . xviii 455

supplementary muscles of . xii 331

the incision for fissure of . v 485

Anxiety, morbid, observations on . xvii 419

Aorta, abdominal aneurism of . xxiv 417

on branches of . . xiv 380

aneurism of the ascending . ix 548

Dr. Geddings on signs of i 160, vii 366

treatment of . . i 161

arch of, deviations of . . xix 487

ascending, aneurism of . . xxiv 416

case of aneurism of . xv 148

bifurcation of, case of . . xxi 471

branches from, deviations of . xix 489

Aorta, bruit de soufflet in, controversy i 458
case of transposition of . . xv 147
cases of aneurism of arch of . ix 571
closure of arteries of arch of . xii 134
coagulated lymph in the . xiv 128
compression of, in flooding . x 274
contracted, case of . . xvi 326
dilatation of, Dr. Geddings on i 160
diseases of, Dr. Geddings on i 157
of orifice and valves of . xvi 323
dissecting aneurism of . . xx 94
exploration of, by percussion . vi 134
inadequacy of valves of . v 272
inadequate valves of, signs of v 272
inflamed, symptoms of, obscure i 159
inflammation of . . . . xxiv 412
inflammatory effusions in . xiv 128
morbid changes in the . xiv 128
obliterated and contracted . xx 225
beyond the arch . . xiii 255
on vegetations in the . . xvi 325
on violent pulsations of the . iv 533
partial redness of lining of . xiv 130
rupture of different parts of . iii 70
ruptured, remarkable case of . iii 70
transposition of . . . . xii 330
sigmoid valves of obstructed, sounds of . . . . i 484
tying of, in animals, effects of xxii 108
ulcerative softening of the . vi 46
unusual conformation of arch of xix 489
vegetations on valves of the . v 600
wound of, cured . . . . vii 254
the arch of . . . . xiii 542
yellowish specks on the . . vi 46
Aortic arch, aneurism of . . xxiv 416
ostium, dilatation of . . xvi 324
orifice, contraction of . . xvi 326
dilatation of . . xvi 324
Aortitis, erysipelatous type of . xiv 129
on the causes of . . . xiv 131
a cause of angina pectoris . v 273
acute, results of . . xiv 128, 130
cases of . . . . . . v 273
treatment of . . . . . v 274
Apartments, ventilation of . . xix 6
Ape, on the arm of the . . . xiii 474
on the worship of the . . x 252
Apes, on the length of arms of . xxiv 80
anthropomorphous, osteology of xvi 373-77
transition in the skulls of . x 252
Aperient medicines, abuse of . . ii 389
on employment of . . x 40, 49
Aperients, remarks on the abuse of v 390, 392
Dr. Burne on the use of . . x 46, 49
and purgatives, remarks on . xiv 333
Aphonia cured by swallowing ice . v 439
sudden, in females . . . v 439
Aphonia, stubborn case of, cured . viii 252
Aphoria or sterility, Dr. M. Hall on xxiii 191
Aphorisms in medicine, Dr. Pauli's xxii 53
on the birth of man . . xix 557
Aphorisms and puns by Professor Marx xix 125
Aphrodisiac, cantharides as an . ii 549
Aphtha, epizootic, in cows . . xiv 400
epizootica, infectious through milk . . . . xiii 47
infectious to man . . xiii 47
symptoms of . . . xiii 47
Aphthæ, attachment of . . . xxiv 424
causes of . . . . xxiv 430
colour of stools in . . . xxiv 428
contagious nature of . . xxiv 431
danger of, in hospitals . . xxiv 432
description of . . . xxiv 427
habitat of . . . . . xxiv 425
microscopical character of xxiv 423, 425
orientales . . . . . xii 516
results of experiments on . xxiv 428
treatment of . . . . . xxiv 433
and muguet, distinction between ii 367
Dr. Naumann on . . ii 367
report on . . . xvii 550
treatment of . . . ii 367
Aphthous fever, epidemic in Holland xii 114
Aponeuroses, peculiarity in . . iv 170-71
the seat of rheumatism . . iii 78
Apoplectic cases, primarily so . iii 318
not primarily so . . iii 319
coma, Dr. Burrows on . . xxii 418
convulsions, Dr. Hull on . xvii 203
Apoplexia intermeningea, remark on xiv 223
sanguinea, case of . . . xxii 387
ex inanitione . . . . iii 325
and asphyxia neonatorum . xx 551
Apoplexy, abdominal in children . xv 248
active practice in . . . xvii 131
and cardiac disease, connexion of xxii 420
and disease of heart . . viii 504
and heart-disease, coincident . vi 144
and hypertrophy of the heart . ii 333
and syncope, not allied . . xxii 416
a variety of remarks on . . i 26
cause of . . . . . xxi 273
cerebral, in old age . . xvii 110
symptoms of . . . xvii 110
collection of cases of . . xx 197
congestive, remarks on . . xiv 56
connexion of with gout . xvii 111, 113
contraction of the limbs in . xx 258
cutaneous, case of . . . viii 552
deaths from, in England . . ix 356
diagnosis of, from coma . . xvii 130
Dr. Abercrombie on . . iii 318
Dr. Bennet on . . . . xii 103
Dr. Watson on bleeding in . xvii 132
dull and blackish blood in . viii 324
frequency of in old age . . xvii 110
from cardiac diseases . . xvii 111
from fright . . . . . xi 235
hard and jarring pulse in . ix 485
in a boy, case of . . . xxiii 302
in children . . . . . xxi 384
infantile, Dr. Kennedy on . iii 550

Apoplexy in infants, remarks on . ii 360

influence of weather on . . x 61

in relation to age . . . xxii 422

in relation to the seasons . xvii 111

in still-born children . . xxiii 298

M. Gay on . . . . . xvi 272

against bleeding in . . xvi 272

meningeal, Dr. Prus on . . xxi 409

mercurial inunction in . . viii 420

observations on . . . xiv 340

of infants . . . . . xvii 553

of newborn infants . . . vii 92

of the eyes . . . . . ix 548

on bloodletting in . . . iii 322

on extravasation of blood in . iii 318-19

on loss of speech in . . xxii 461

on no morbid traces after . iii 327

on purgatives in . . xvii 132, iii 322

on stimulants in . . . xii 103

particular class of cases of . iii 11

passive, in children . . xxi 385

prevalence of in Milan . . x 425

prevention of, by bleeding . xvii 112

pulmonary, Dr. Graves on . xvi 248

remarks on . . . viii 333

seat of . . . . . xv 88

treatment of . . . xvi 249

renal, M. Rayer on . . xv 478

secondary, case of . . . iii 320

sequelæ of, importance of . xxii 424

serous, objections to the term . iii 312

statistics of . . . . . xx 198

traumatic, on bleeding in . xvii 200

treatment of . . . iii 322, xii 103

Dr. Burrows on . . xxii 423

use of emetics in . . . xvi 272

with cardiac lesions, tables of . xxii 421

Apothecaries' act of 1815, effect of xiv 409

and druggists . . . xiii 212

company, and Latin . . i 519

certificates, 1835-6 . . iii 292

in Belgium . . . . . i 608

in France only sell drugs . i 495

in Norway . . . . . iv 546

licenses, Irish, number of in ten years . . . . . . i 610

Sir J. Clark on . . . xv 530

society of, report of examiners of ii 606

weights, relative value of . i 609

Apothecary of the United States .. ii 593

Appendix cæci, perforation of . iv 149

vermiformis, hernia of . i 583

perforation of . . xx 222

of cæcum, perforation of . ix 452

Apples, easily digested . . . xvii 32

Appetite in relation to respiration . xix 10

Appointments, royal, in Scotland . v 626

Apricot tree, magnetising of an . vii 321

Aqua colorata, hospital use of . xxiii 607

phagædenica, burns cured by . i 581

Aqua picea, use of in psoriasis . xxiv 48

Aqueducts of internal ear . . viii 70, 80

Aqueous capsule of the eye . . vi 205

discharge after labour . . xix 356

humour, theory on the . . x 13

membrane of eye, disease of . xi 70

vapour favours putrefaction . ii 397

Aquo-capsulitis, Mr. Bedford on . xvi 323

Arabian drugs derived from India . xxiii 528

horses, the breeding of . vii 381

period of medical history . xxiii 95

school of medicine . . xxiii 527

the most perfect human skull xxiv 455

Arabistæ of the middle ages . . xviii 37

Arachnida, digestive apparatus of the i 538

division of, by Latreille . . i 537

Arachnitis, actual cautery in . xxii 397

at the base of the brain, case of xxii 396

cerebro-spinal, report on . xxiii 302

complications of . . xxii 399, 405

death in sleep from . . xxii 407

dense false membrane in . iv 38

fatal complications in . . xxii 407

general notanda of . . . xxii 404

in renal dropsy, treatment of . ii 230

morbid exudations in . . iv 39

on the products of . . iv 39

Arachnoid, Dr. Hodgkin on the . iv 38

extravasations of blood into cavity of . . . . xxiii 410

ganglionic character of . xxiv 324

hemorrhage, sub- and intra- xxi 410, 411

morbid anatomy of the . iv 40

new views of the . . . xxii 407

peculiarities of the . . xiv 463

suppuration of, case of . . xiv 227

Aram, Eugene, Dr. Inglis on the skull of vi 581

Archæus of Van Helmont . i 385, xvi 406

Architecture and ventilation, relations of . . . . . xix 20

Archives de Médecine . . . i 225

North American, of Medical Science i 233

Arctic regions, on food in the . xiv 508

Area germinativa, in blastoderma xvi 547

pellucida, in the embryo . xv 266

Areola, as a sign of pregnancy iii 405, iv 184, 452

changes of by uterine disease . iv 453

changes of colour of the . x 114

colour of in pregnancy . x 114

cuticle of the . . . x 114

description of the tubercles of x 114

diagnosis of pregnancy by the ii 403

in pregnancy, description of . iv 452

mammary, venous circle of . vii 240

of breast, anatomy of . . x 114

of nipple as a sign of pregnancy i 100

darkness of . . xxiv 70

papillæ of the, use of . . x 114

rete mucosum of the . . x 114

situation and size of . . x 114

state of the, as a sign of pregnancy iii 140

Areolar pultaceous cancer . . xxi 426

tissue, observations on . . xxi 494

Areskin, Dr., his honours in Russia i 604

Aretæus aware of blood in arteries iv 492
on diseases, translation of . iv 490
Argel leaves, Dr. Pereira's account of xiii 81
Argenti nitras in diseases of skin . v 287
in female gonorrhœa . iv 540
internal use of . . iv 116
on blue skin from . . iv 116
Argentil, Marquis d', his prize . xxii 233
Argentine solution into larynx . xxiv 501
Argentum nitratum in follicular disease xxiv 497
Argill, its use in infantile cholera . i 257
Argumentation, effects of the love of xxiii 162
Arians, or Indo-European nations . xxiv 456
Aristotle, account of . . xxiii 92, 99
anatomical discoveries of . xv 107
cultivated comparative anatomy i 380
his physiology . . . i 381
philosophy of . . . v 82
philosophy proper to . . xxiii 99
the founder of embryology . i 380
Arithmetic, on teaching idiots . xxiv 12
and algebra . . . . xii 3
Arm-baths, use of in croup . . xii 246
Arm, bent, division of tendons for . ix 230
of child, in labour, on amputation of ii 526
presentation, case of . . xiv 582
of, three times . . xx 533
report on . . . xvii 537
Armstrong, Dr. J., his death and character . . . . . i 67-68
his early education . . i 35
his illness . . . . . i 63
his lectures, by Mr. Rix . . i 34
his life, by Dr. Boott . . i 34
his manner as a lecturer . i 57
his sarcasms on Cullen . . i 60
his success in London . . i 53, 56
his work on typhus . . i 42
on climate, &c. . . . xviii 221
on puerperal fever . . . i 39
opposite opinions of him . i 34
rejected by the College of Physicians . . . i 54
rhapsodies of . . . i 59
Armstrong, J., his economy of love v 443
Armies, formation and economy of xxi 166, 171
Army and navy, bowel complaints in xv 16
comparative health of . xvii 313
comparative mortality in xvii 316, 328
diseases of liver in . . xv 16
diseases of lungs in . xv 11
medical observation in . xxiii 107
phthisis in . . . xv 11
relative health of xv 1, 13, 17, 18
various diseases in, relative prevalence of xvii 316-28
condition of the soldier in the xvii 313
health of United States . . xvi 34
imprisonment in, objections to xxiii 167
medical museum . . . vii 225
drawings from . . vii 224
medical officer, duty of . . xx 202
Army medical officer, reports of . ix 68
staff of Peter the Great . i 604
statistics of the . xi 364, xv 1
objections to flogging in the . xxiii 166
on the field carriage of . xxii 225
on the medical hygiene of . xix 377
surgeon, on his responsibility xxiii 165
tents, old, fever from . . i 377
Arnica, formulæ of . . . ii 212
in paralysis of auditory nerve . iii 85
properties and use of . . ii 211
use of, in paralysis of bladder xix 374
Arnold Dr., on development of the iris i 551
his critique on Dr. Hall . . xvi 174
his Jamaica Journal . . iii 193
on origin of first cervical nerve i 550
on particular nerves . xi 517
on the reflex theory . xvi 158
on vomiting and emetics . xv 60, 74
Arnold, F., on human physiology viii 476
Arnold, W., on pathological physiology viii 476
on the spinal nerves . . xix 558
on the laws of the living body . v 75, 90
Arnold, W. and F., on cartilage and bone i 231
Arnott, Dr. J., his fluid urethral dilator xiii 319
his urethral dilator . . xii 407
his useful inventions . . xxi 499
on dilating the urethra . . xiii 318
on stricture of urethra . xii 388, 406, xiii 312, 327
on therapeutical inquiry . . xxi 498
Arnott, Dr. Neill, his great liberality v 547
his useful inventions . . xxi 218
his water bed, remark on . ii 64
on compression in cancer . xxi 221
on warming and ventilating . v 546
Arnott, Drs., their invention for stone xii 406
Arnott, Mr. J. M., his case of osseous tumour . . . xi 458
his Hunterian oration . . xv 536
on wound of tibial artery . xxiv 323
Arnott's elements of physics, remarks on ix 529
Arntzenius, Dr., on the urethra xiii 312, 326
on suicide . . . . v 369
Aromatic wine of the French codex x 403
Arrangement in books, remarks on vii 117
Arrot, Dr., on fever in Dundee . xviii 179
Arrow-root, adulteration of . . xiii 78
diagnosis of purity of . . xiii 78
effects of feeding infants on . xxiii 498
fecula, extraction of . . xiii 77
properties of . . xiii 77
Arsenical and anatomical stains . xvi 76
poisoning, bleeding in . . xi 45
Dr. Nicolai on . . xvi 75
lime-water in . . iv 360
Mr. Taylor on . . iv 358
on pain in . . . iv 359
on thirst in . . . iv 360
time of death in . . iv 360
stain, Mr. Marsh on . . xiv 123
stains, characters of . . xi 40

Arsenical, stains resembling . xiii 184, 193

Arsenic, absorption of, into the blood xi 37, 45

action of . . . . . viii 569

analysis for detection of . iv 360-1

and quinine in fevers . . xiv 47, 49

as given by murderers . . iv 364

case of poisoning by xii 564, xiv 122, xvi 322

cases of poisoning by viii 568, xviii 541, xix 375

caution on protracted use of . viii 360

chemical evidence of . . xx 330

cigars of, in phthisis . . xii 249

copper test for . . . xiv 124

advantages of . . xvi 277

objections to . . . xvi 276

curious fact relative to . iv 363

detected in a patient's blood . xi 45

detection of . . . . . ix 58

in liquids . . . xiv 258

treatises on . . xiii 183

Dr. Fresenius on detection of xviii 537

Dr. Hunt on the exhibition of xviii 68

Dr. Shearman on detection of xviii 539

effect of boiling it in water . iv 363

experiments on an antidote to i 594

on antidotes for . . iv 234

form of administering . . vii 152

history of, as a poison . . xx 329

how taken by suicides . . iv 363

hydrated oxide of iron an antidote to . . . . i 572-3, 594

impediments to solution of . iv 364

improved antidote for . . xvi 154

inflammation caused by . . xiv 125

in broth, gruel, &c. . . iv 364

in cemeteries, remarks on . xiii 196

in zinc and sulphuric acid . xiii 192

in the body naturally or not xviii 541-2

in the soil of graveyards . xi 51-2

its solubility in water, &c. iv 358, 361

its use in tic douloureux . xviii 60

its use in uterine diseases . vii 151

loss of, from bodies by drainage xi 51

medicinal action of . . xiv 48

minute doses of, in fevers . xiv 47-8

Marsh's apparatus for . . iv 360

hydrogen test for . . viii 360

process for detecting . xi 38, 39

test for . . . . . xvi 76

new mode for using xviii 536

M. Orfila's experiments on . xi 38, 44

apparatus for detecting . xi 39

process for detecting xi 40, 44, 47

on Marsh's test for xvi 275, 278, 281

naturally in the body . . xi 45, 49

whether . . xx 331

naturally in bone . . . xi 49, 52

denied . . . xii 347

in muscles or not . . xi 50

new copper test of . . xvi 275-82

new mode of prescribing . xiv 47-8

of detecting . . viii 573

new processes for detecting . xv 251

Arsenic, nitric acid for detecting . xi 46-8

not in the body naturally . xvi 77

not in the bones normally . xiii 195

not normally in the body . xiii 184, 195

often in sulphuric acid . . xviii 542

on antidotes for . . . ix 57

oxide of iron as an antidote to viii 573

poisoning by . . . xi 37

case of . . . . iii 528

cured . . . . vii 563

cases of . . i 572-3

report on . . . xviii 536

popular use of, as a tonic . xix 375

purity of reagents for . . xi 45

reckless sale of . . . xviii 551

Reinsch's new test for . xvi 275, xviii 541

removal of, from the system . xiii 185

recovery from large dose of . xiii 572

saturated solution of . . iv 363

separation of from antimony . xvi 77

sesquioxide of iron antidote of x 287

smallest fatal dose of . . xii 342

taste of . . . iv 364, ix 57

tests for . . . . . v 213

treatment of poisoning by . viii 575

various means of detecting . xviii 537

ulceration and gangrene from . iv 360-1

use of, in chorea . . . ix 269

in diseases of the skin . xxiv 280

nitre for detecting xi 46, xiii 194

in plethora . . . v 305

what dose of, poisonous . viii 570

with mudàrr, formula of . xii 425

Arsenious acid, reducing agent for . xx 328

Arsenite of copper, on poisoning by xviii 551

Art, ancient and modern compared xviii 444

and nature in the cure of diseases xxiii 594

and science, disquisition on . vi 103

medical, on the imperfection of iii 200

on empirical and scientific . xxii 239

scientific or empirical . . vi 104

Sir J. Herschel's definition of vi 106

Arts, ancient, recent discoveries of xxiii 521

health in, effect of civilization on xviii 243

of design, use of anatomy in xviii 442

Artemisia santonica, sugared . xiii 87

root, sedative properties of . xiii 346

Arteria innominata, position of . xix 105

Arterial circulation, report on the xix 262

coats, softening of the . . i 158

compressor of Segnoroni . xxii 111

elasticity and contractility . xviii 70

hemorrhage, suppression of . xii 441

inorganic murmurs in anæmia viii 459

linings on red stains in . . xiv 129

system, on changes in the . iii 220

on the pathology of the . iii 220

tubes, relative capacity of . ix 421

various shapes of . . vi 45

tunics, Malgaigne on . . x 358

Arteries, agency of, in circulation . viii 172

abnormal sounds of, four species i 456

anastomosis of . . . xxii 104

Arteries, anatomy of, observations on xiv 378
and circulation, remarks on . viii 514
and veins, elasticity of . . xxiv 411
structure of . . . xxiv 410
atheromatous, in old age . xxii 422
deposit in . . . xxiii 70
matter in . . . vi 47
bleeding, on pinching . . vi 157
torsion of . . . vi 157
bruit, musical, of . . . i 456
on ronflement du diable of i 456
caliber of the . . . vi 44
catgut ligatures for . . xiv 24
causes of the various sounds in i 457
continuous bellows-sound of . i 456
contractile power of . . viii 334
cutting short ligatures on . vi 159
cylindrical form of, rare . vi 45
deligation of, Lisfranc on . xiv 21
development of . . . . xix 590
different methods of tying . xxii 96
diseases of . . . . . xii 114
division of coats of, by ligature xxii 95
Dr. Beck on the ligature of . iv 154
Dr. Billing on the . . v 201
effects of ligature on . vi 158, xxii 94
experiments of J. Hunter on viii 459
elasticity of the . . . viii 172-3
exudation in . . . vi 45
fatty degeneration of . . xviii 366
ganglionic influence on the . viii 174
general structure of . . xiv 284
healing of wounded . . xii 441
helicine, of Müller . . xiv 295
Hunter on the functions of . xiv 285
on the structure of viii 171, 173
Hunter's view of action of . viii 172
inner coat of, not vascular . xxii 90
ligature and torsion of . . xxi 287
ligatures for, experiments on . xxii 91
local and remote ligature of . iv 155
M. Bizot's researches on . vi 41
mediate and double ligature of xxii 97
morbid closure of . . . xii 134
muscular compression of . v 147
fibres of . . . viii 173
muscularity of . viii 172-3, xxiv 411
neuralgia of . . . xii 114
normal sounds of the . . i 455
obliteration of . . . v 564
of corneal conjunctiva, Dr. Römer on ii 235
of extremities, inflammation of xxiv 412
ordinary intermittent bellows-
sound of . . . . i 456
ossification in . . vi 46, 47, 48
outlines of the . . . xxii 231
pathology of . . . iii 69
Prof. Porta's work on . . xxii 89
Quain's anatomy of the . xi 210
on the . . . xix 486
great work on . . xxii 556
red coloration in . . . vi 45
redness of after death . . ix 225
of internal coats of . i 250
Arteries, remarks on wounded . iv 159
report on . . . . xxi 551
rules for remote ligature of . iv 155-8
size of branches and trunks . xiv 574
softening of the coats of the . i 158
sounds audible in . . i 455
structure of the coats of . x 551
surgical anatomy of the . viii 538
sympathetic nerves in . . xii 114
structure of . . . x 358
termination of, in veins . xiv 295
torsion of . . . . xxii 101
modification of . . viii 559
tying above and below . . x 54
main trunks of . . x 102
vital dilatation of . . xxii 105
umbilical, inflammation of . xiii 528
white patches in . . vi 45-6
effects of . . . vi 46
wounds of the palmar . . iv 157
wounded, slight pressure in . xi 309
Arteriotomy, Dr. Pancoast on . xx 45-7
Arteritis after ligature . . xxii 100
M. Bonetti on . . . viii 556
Mr. Crisp on . . . xxiv 411
treatment of . . . xxiv 413
Artery, axillary, obliteration of . xiv 254
basilar, peculiar structure of . vii 574
brachial, obstruction of . iii 242
carotid, cases of ligature of . xxiv 323
compression of, in amputation xiii 172
compressor, M. Malapert's . xvi 64
femoral, case of rupture of . iii 122
cases of wounded . iv 155-6
ligature of . . . vii 153
humeral, wounds of . . xx 48
on ligature of external iliac . vii 154
posterior tibial, ligature of . xxiv 323
pulmonary, laceration of . xiv 547
subclavian, case of ligature of xxiv 323
superior thyroid, tying of . xxii 50
Arthralgia or lead rheumatism . x 447
Arthritic anomalous ailments . xiv 413
hydrothorax of old age . xvii 115
tubercular disease of brain . xvii 114
Arthritis, acetate of morphia in . ix 252
Articular cartilage, suppuration
of . . . . . . xiv 545
cartilages, diseases of . x 338
vascularity of . . xxiii 71
vessels in . . . xi 452
cavities, on accidental . . xviii 82
surfaces of joints, excision of . xxii 370
Articulata, fibres of, reflex action in xvii 184
ganglionic tract in the . xvii 184
Mr. Newport's neurology of . xvii 183
nervous cord in the . . xx 491
system of the v 494, ix 102, xvii 181-2
two nervous tracts in the . xvii 182
on the ventral cord of . xxi 460
sensori-volitional system of . xvii 184
structure and growth of . xx 487
Articulation, deranged, source of . iv 379
diseased spino-occipital . . v 278

Articulations, Mr. Blackburn on the . x 330

surgery of . . . . . xiii 169

Artificial anus, operation for . xiv 231, 250

treatment of . . . xxiii 27

various treatises on . . xviii 452

premature labour, case of . v 578

pupil, formation of . . xxiv 369

treatise on . . . xxiv 364

Artisans, diseases of, M. Chevallier on i 282

Artist, the "nascitur, non fit" . xviii 451

Artists, abuse of anatomy by . xiv 528

Ascaris, development of ova of the . xx 417

Ascherson, Dr., on oil-globules . xiv 442

Ascites, chiefly caused by morbid liver iii 436

cured by large blister . . xxi 370

dissection of, cases of . . iii 438

Dr. Seymour on . . . iii 436

extraordinary causes of . . xxi 369

from tuberculated peritoneum iii 438

graphic description of . . iii 437

hydriodate of potash in . iii 438

in children, paracentesis in . xxi 471

means of curing without tapping ii 230

on acupuncture in . . v 279

on tapping in . . . iii 437

remarkable case of . . xx 241

serum tinged with blood in . iii 438

state of the liver in . . iii 437

sudden subsidence of . . iii 438

treatment of . . . iii 437

various rubefacients for . . ii 230

venosus of old age . . xvii 116

vomiting of green liquid in . iii 438

Asclepiadæ, labours of the . . xvii 458

of the Greeks . . xvii 456, 458

Asclepiades, Aristophanes' jokes on the xxiii 90

of Bythinia, account of . xxiii 102-3

Ashes, commercial value of . . xxi 339

Ashwell, Dr., his obstetric cases iv 369, vii 451

on chlorosis . . . iii 156

on diseases of women xi 174, xix 344, xxiii 239, 612

on female diseases . . xvi 512

on occlusion of the uterus . ix 382

on producing premature labour. ii 199

on undue lactation . . xii 328

on uterine diseases . . vi 71

hemorrhage . . vii 450

remarks on his style . . iii 157

Asiatic cholera, effect of civilization on xviii 253

Asclepias gigantea or mudàrr, use of xii 425

Aspalasomus genus of monsters . viii 6

Asparagine, chemical composition of xiv 517

Asphaltum, oil of, in consumption . xii 432

Asphyxia, and apoplexia neonatorum xx 551

artificial respiration in . . v 277

case in relation to . . v 583

congenital, heat and cold in . xvii 180

Dr. Carpenter on . . . xii 112

Dr. Hall on . . . . xiv 198

Dr. Harlan on death from . ii 519

effect of, on capillaries . . xvii 493

from abscess, fatal . . xiii 230

Asphyxia, from carbonic acid . xii 264

gas . . vi 242, xiv 197

state of the blood in . . xvii 433

from coal gas, cases of . . xv 252

from section of recurrent nerves iii 29

from shortening of the funis . ii 359

inflation of oxygen in . . xxi 270

in infants, treatment of . . v 277

in new-born infants, causes of ii 359

classed . . . . ii 359

medical jurisprudence of . ix 49

Mr. Erichsen on . xxi 265, 555

movements in, Dr. Hall on . xvii 180

on death by . . . v 175

on the blood in lungs in . ii 423-4

on cause of death in . . v 104

rigidity after death from . ii 425

state of circulation in . . xii 278

successive effects in . . xii 278

three opinions on . . . xxi 266

neonatorum, remarks on . viii 474

report on . . . xxiii 300

treatment of . . . viii 52

two kinds of . . . iii 443

secondary, from opium . . xviii 557

of infants . . . xvii 180

remarks on . . . xiv 198

Asphyxial phenomena, duration of . xxi 268

Assafœtida, experiments on . xiii 575

its effects on the heart i 264, iii 510

plaister, a formula for . . iii 510

Assassin, derivation of the term . xxiii 218

Asses eaten by the Tartars, &c. . xvii 16

Assimilating organs, vast hypothesis on xi 333

Assimilation, action of water in . xi 331

cultivation of . . . xvi 217

destructive, Dr. Prout on xi 333, xvi 485

diseases of the organs of . xxiii 304

Dr. Carpenter on . . . xv 269

Dr. Prout's hypothesis on . xi 335

formative, Dr. Prout on . xi 333

morbid oleaginous . . xi 355

of ingesta, rate of . . x 101

primary, Dr. Prout on . . xvi 480

process of reduction in . . xi 331

report on . . . . xvii 261

secondary, Dr. Prout on . xvi 484

two classes of . . . xi 331

use of the lungs in . . i 246

Assimilative process, remarks on . i 389

Association, the British, reports of i 368, ii 594

meeting of the . . iv 567

remarks on the . . iv 551

Medical . . . iii 289

of General Practitioners. . xix 614

of ideas, remarks on . . xix 311

Irish Medical . . . x 292

Medical and Surgical . . iv 551

of Belgium . . . i 606

of Prussia in Berlin . ii 582

of Vienna . . . xix 360

Provincial, on influenza . . vii 95

eastern branch . . ii 300

Association, Provincial, Transactions of i 529, ii 300, 601, iii 159, iv 551, 560, vi 1, viii 303, ix 216, x 293, xvii 92, xx 508, xxii 319.

Associations, medical, in America . iii 273

Assurance companies, on fees from xiii 578

offices, glaring errors of . . iii 54

of this country . . iii 53

on medical fees by . . iii 54

ruin from some . . iii 53

tables of mortality of . iii 53

societies, certificates to . v 311

tables of the Equitable Society . iii 40, 54

Asthenopia, Dr. Mackenzie on . xx 517

Asthma, arthriticum of old age . xvii 114

gonorrhoicum of old age . xvii 114

grinders', morbid anatomy of . xiv 580

objection to the term . xviii 304

hay, case of . . . . xx 245

Indian, remedy for . . xix 74

in relation to the par vagum . iii 28

juice of the poke in . . xix 25

Koppian, an improper title . ii 240

nervous or spasmodic . . xi 264

on a warm climate for . . xii 164

on counter-irritants in . . xvi 246

on wheezing in . . . . xi 265

or rot of grinders . . . xxii 320

phytolacca decandria in . . xxi 372

remarks on the treatment of . xix 25

spasmodic, treatment of . . xvii 420

thymic, an improper title . ii 240

cases of by Dr. Kopp, &c. ii 237-8

Dr. Hirsch on . . ii 237

history of . . . ii 238

nature of . . . ii 239

objections respecting . ii 240

pathognomonic symptom of ii 239

prognosis in . . . ii 240

the same as laryngismus stridulus . . . ii 240

treatment of . . . ii 240

cupri sulphas in . . i 258

of the Germans . . i 258

urinosum of old age . . xvii 114

use of creosote in . . ii 169

use of quinine in . . . xv 239

Asthmatic affections of old age . xvii 114

Astragalus, dislocated, reduction of xviii 132

leaving . . . . . xviii 133

dislocation of, rare kind of . xviii 125

excision of, Mr. Turner on . xviii 133

on dislocations of xviii 124, 130, 565

on extirpation of . . . xviii 124

Rognetta's experiments on . xviii 131

rotation of, on its axis . . xviii 132

Astringents, in uterine hemorrhage, when proper . . . ii 515

often injurious in uterine hemorrhage . . . . ii 515

with narcotics . . . xvi 200

with tonics . . . . xvi 200

Astronomy, influence of the study of ix 421

built on calculation . . xii 4

Asylum, for the insane of middle classes xx 479

insane, at Berlin, bad . . i 500

lunatic, on an improved plan . i 308

music in . . . xv 282

medical, superintendent of an xxiv 164

Asylums, admission of lunatics into xix 327

dress of nurses in . . xxiv 161

lunatic, abuses and defects in . xix 323

boys as attendants in . xx 480

cruel economy in . . xxiv 159

Dr. Conolly on . . xxiv 156

duties of physicians of . xix 601

economy, &c., of . . xxii 357

government of . . xix 600

in France . . . xix 594

management of . . ix 144

minute attentions in . xxiv 158

on diet in . . . xxii 358

on scenery around . . xxiv 157

recoveries in . . xxii 360

remarks on . . . xxi 464

various kinds of . xix 319, 323

private, existing evils in . . xix 613

lunatic, of Paris . . xix 608

on English and French . xix 612

visiting justices of . xix 321, 326

Asymmetrical development . . xiii 557

Atavism, on the law called . . viii 487

Atelectasis of Dr. Joerg . . vi 66

Athletæ club, account of the . xvi 134

Atheromatous arteries in old age . xxii 422

deposit in arteries . . . xxiii 70

Atlas, fracture and displacement of iv 141

singular case of fractured . vi 161

Atlas of cutaneous diseases, M. Rayer's ii 157

of plates, Dr. Mouatt's . . xxii 227

Atmosphere, density of, Dr. Dunglison on i 345

Dunglison on dry and moist . i 347

how purified . . . . xx 348

its influence on health . . i 345

miasmatic, mortality in . . xviii 501

moist, a cause of diseases . xx 102

rarity of, effects of . . . vii 69

vitiated, evil effects from . xx 429

vitiation of, report on . . xviii 498

Atmospheres, artificial . . . xix 15

Atmospheric air, on moisture in xix 8, 11, 12

pressure, effect of . . . vii 69

influence of . . . xxiv 98

Atom, definition of an . . . xi 139

Atomic theorists, creed of the . xi 130

theory of Dalton . . . xi 130

Atoms and organic molecules, essay on xi 137

physical and chemical . . xi 138

ultimate, doctrine of . . xi 138

Atresia of the vagina, case of . xiv 563

Atrophia ablactatorum, report on . xx 558

Atrophy, caused by compression . ii 461

cirrhose, of liver, described . ii 462

Dr. Carswell on . . . ii 460

from contractile fibrous tissue ii 462

from diminished blood . . ii 461

from loss of nervous influence ii 463

from want of functional exercise ii 465

Atrophy in dyspepsia . . . ii 463

in hypochondriasis, Whytt on ii 464

in painters' colic, cause of . ii 463

in paralysis . . , . ii 463

local forms of . . . ii 461

of abdominal viscera . . ii 462

of bone, concentric . . iv 152

eccentric . . . iv 153

from diminished blood . iv 152

in fractures . . . iv 153

Mr. Curling on . . iv 152

of brain in children . . xxi 389

in insanity . . . ii 461

of face, new form of . . xxiv 43

of heart, cases of . . . ii 336

case of, from Laennec . ii 465

causes of . . . ii 336

diagnostics of . . vi 134

of leg, case of . . . xvi 267

of lung, from compressed bronchia ii 465

of lungs from emphysema . ii 461

of mammæ, on cessation of menses . . . . . ii 465

Attendants in lunatic asylums, on . xxiv 163

Attention, effect of, on the nerves . xvii 409

mental, effect of, on organs . viii 492

Attenuations, homœopathic . . xxi 229

Attitudes and exercises, Dr. Kilgour on i 356

of idiots, correction of . . xxiv 13

Attraction, adhesive, Dr. Daubeny on xi 144

capillary, Matteucci on . . xxiii 382

cohesive, in chemistry . . xi 144

Auburn prison (U. S.), system in . xii 500

Auditory nerve, functions of the . viii 87

M. Flourens on . . . xvi 371

papillæ of the . . . vi 420

Augusta arsenal, in America . . xvi 40

Auricle, dilatation of right, diagnosis of vi 134

Auricles, systole of, a notable impulse in i 448

undulatory motion in . i 448

Auricular opening, narrowing of . xx 254

Aurist, remarks on the name . viii 90

Aurists, English, Kramer's strictures on iii 96, 98

Auscultation, advantages of mediate iv 295

adversaries of . . . ix 300-40

and auscultators . . . viii 467

and percussion, Dr. Cowan on . iii 189

Barth and Roger on . . xiii 518

by ear, rarely best . . iii 188

cerebral . . . vii 265, xx 255

credit of England in . . iii 184

deceptions in abdominal . viii 371

Dr. Bennet on . . . xiv 180

Dr. Bowditch's aid to . . xxii 209

Dr. Breventani on . . v 167

Dr. Günzburg on . . . xxii 232

Dr. Hughes's manual of . . xxi 281

Dr. Mer. Laennec on . . iv 285, v 423

Dr. Newbigging on . . xiv 173, 180

Dr. Phillipp's work on . . iii 185

Dr. Seymour a derider of . xxiv 139, 145

Dr. Skoda on . . . xiv 173

Dr. Wolff on . . . v 215

experiments in . . . v 590

Auscultation, great use of in pregnancy iv 457

ignorance of students of . ii 474

immediate, opinions on . . iv 295

in dilatation of stomach . . i 487

in diseases of the ear . . iii 96

in infantile pneumonia . . vii 80

in pregnancy, authors on . iii 406

value of . . . xiv 208

manual of, by Raciborski . i 479

M. Fournet on . . . xiii 220

neglect of, in Germany . . iii 185

not illimitable . . . i 479

not valued in Italy . . i 498

obstetric . . . . viii 365

history of . . . i 86

obstetrical . . . . vii 230

of children . . . . xii 357

of the heart . . . . xx 180

of the voice, mode of . . ix 329

on artificial . . . . vi 532

practice of, on corpses . . vi 532

researches in . . . ix 299

Auscultators, young, cautions to . xvi 492

Auscultic paradox, Dr. Hudson on an i 488

Auscultory experiments with sponge ix 302

Auscultatory signs, principle of . iv 295

Auscultology, Dr. Skoda's new . xiv 175

Austral Asia, animals and plants of iii 370

Australasian native tribes . xxiv 470, 471

Australia, on the languages of . xxiv 471

Australian children, intelligence of xxiv 472

Austria, history of foundlings in . xxii 338

statistics of calculus in . . iii 254

vaccination in . . . ii 280

Austrian empire, state of medicine in i 1

medical annals, German . i 219

Authority, on medical and surgical xxi 285

on unbounded respect for . xii 53

Authorship, medical, remarks on . xxi 441

Authors, medical, misdoings of . xxi 499

on the study of the older . ix 107

on young medical . . . ii 490

Automatic action, Prochaska on . xxiii 212

actions, secondary . . v 518

Autoplasty, or plastic surgery . vii 386

Autoplastic operation on the eye . xi 219

Autrefois convict, plea of . . v 174

Aversion and desire, Unzer on . xxiv 191

Aversions and desires, action and nature of . . . . xxiv 198

Aveyron, the savage of, and Itard . xxiv 20

Avicenna, his midwifery . . xiv 83

on smallpox . . . . vi 483

Awakened, suddenly, murder by the x 152-65

the half, murder by the . . x 165

Axiom, a great practical . . xvii 500

Axillary dislocations . . . xii 455

artery, obliteration of . . xiv 254

wound of . . . xiii 564

Aylesbury, topography of the vale of x 464

vale of, diseases in the . . x 464

Ayr, odd doctrines in . . . xv 540

Ayur Veda, account of the . . xxiii 530

Azores, climate of the . . . xii 172

Azores, Dr. Bullar's account of the . xii 224

Azote, assimilation of, by plants . xi 440

in the stomach, origin of . xi 333

in the system, sources of . xvi 483

source of nutritive . . xi 440

the nutritive principle of plants xiii 487

Azotized materials of organization . xvi 283

Azoturia, nature and treatment of . vii 160

BABBAGE's calculating machine . xix 161
BABINGTON, Dr. B. G., on epilepsy xii 340
on albuminous fluids . . vi 74
on chorea . . . . . xiv 134
on distended gall-bladder . xvi 320
BABINGTON, Mr., on British botany xvii 248
BACHERACHT, Dr., his publications i 606
Back, division of muscles of the . xxi 332
pain of, in intermittent fever . x 557
stay, a simple and efficient . xii 184
BACON, bold and precise style of . vi 99
on science in his day . . xxiii 106
on the advancement of learning vii 116
quotation from, on Great Place xxiv 598
Baden-Baden, mineral springs at xiv 346
BADHAM, Dr., on insect life . . xxi 494
on insects . . . . . v 545
BAER on development of the embryo i 238
on the ovum . . . . . ix 1
Bafureira of Cape de Verd, account of v 563
Bagnères de Bigorre, baths at . xiv 353
de Luchon, spring at . . xiv 347
BAGSTER, Miss, her legal case . x 155
medical evidence on . xvi 86
remarks on the case of . xix 35
Bahama islands, climate of . . xii 172
Bahamas, health of troops in the . viii 214
medical statistics of the . viii 214
BAILLARGER, M., on the brain . x 253
BAILLIE, Dr., eulogy of his character iv 191
on morbid investigation . . ii 456
BAIRD, Mr., on excision of elbow-joint vi 581
Bakers, probity of transatlantic . v 410
Baking of food, remarks on . . xvii 37
Balance, in physiology, use of the . v 332
of functions, disputed . . xvi 441
Balancement of organs, law of . viii 11
Balanitis and balano-posthitis . x 389
and gonorrhœa, diagnosis of . x 389
description and case of . . x 389
or external gonorrhœa . . xii 370
secondary symptoms from . xii 370
treatment of . . . . . x 390
BALBIRNIE, Dr., his absurd style . iii 176, 178
on diseases of the womb . iii 175
BALDERSON, Sir A. Cooper's servant xvi 130, 133
BALFOUR, Dr., his report on homœopathy . . . . . xxii 567
on the Vienna school, and homœopathy . . . . xxiii 607
BALLINGALL's, Sir G., military surgery . . . ix 243, xx 202
on military schools . . xvii 246
BALLING, Dr., on mineral waters and baths . . . . xiv 309
Ballota lanata, account of the . i 567
in rheumatism, &c. . . i 566
BALLY, M., on the use of strychnine vi 225
BALMANNO, Dr., of Glasgow, dinner to i 307
Balsam, copaiba, by enema in gonorrhœa i 578
Gurjun, account of . . xvi 356
of copaiva, Mr. Pereira on . xiii 67
of copaiba, new mode of giving ii 243
BALY, Dr., his Müller's Physiology v 75, 77, ix 244
on dysentery . . . . xxiv 333
on the mortality in prisons . xxiii 411
Bandage, the permanent immovable iii 515
starched, gangrene from . viii 558
Bandages of India rubber . . xii 252
Bandaging in mortification of toes iv 259
new system of . . . xii 252
BANG, Dr., his poem on pathology ii 301
Dr. Ludvig, his praxis medica ii 297
Sidhee, Subjee, or Indian hemp x 225
BANKS, Mr., on medical etiquette . x 215
Barbadoes, diseases of the lungs in . viii 219
Barbel, eggs of, emetic . . . xv 73
Barber, curious case of suicide by a vi 171
Barber-surgeons of Germany, history of xiv 404
BARDSLEY, Dr., his introductory address . . . . i 203, 206
on homœopathy, &c. . . vii 540
retrospective address . . vi 380
Barèges, mineral water of . . xiv 347
Baribice, villages in the valley of . viii 113
BARING, Dr. OTTO, on fungous testicle i 469
Barium, chloride of, in white swellings xi 529
in scrofula, Mr. Phillips on . xxii 152
BARKER and CHEYNE on fever in Ireland (Appendix to vol.) xi 46-9
BARKER, Dr., his cases of aneurism xxiii 416
Bark, excellent preparation of . xvi 200
Barking from pressing the par vagum xiv 133
BARKOW, Dr., on hybernation . xxiv 408
Barley, nutritive degree of . . xvii 19
BARLOW, Dr., account of his writings xviii 283
his address . . . . viii 604
at Bath . . . . vi 570
obituary of . . . . xviii 281
on albuminous urine . x 301, 327
diabetes . . . . xii 334
disease of the kidney . . xvi 308
diseases in early youth . xvi 326
gout and rheumatism . vi 370
ovarian tumours . . iii 165
perforation of the lung . ix 394
the Bath waters, &c., noticed iv 251
the causes of disease . . vi 201
the laws of tubercles . . xii 348
select clinical reports by . xxiii 419
sketch of his life . . . xviii 281
BARLOW, Rev. Mr., on intellectual philosophy and physiology . xv 535
on self-control . . . xvii 245
BARLOW, Mr., his case of tumour . xx 99
BARNARD, Mr., on hydrocephalus . ix 230

Barometer in relation to health xxiv 99, 113
Baron, Dr., his life of Dr. Jenner vi 477
on smallpox and cowpox . vii 295
Barracks, Major Tulloch on roomy viii 225
for soldiers, on the site, &c., of xix 383
Barras, Dr., absurd etiology of . xviii 209
almost ludicrous error of . xviii 208
on cancer of the stomach . xviii 208
Barrier, Dr., on diseases of childhood xv 320
Barry, Dr., criticism on his views xvi 522
his embryological researches . xv 263
on embryology . . . ix 2
on fibre . . . . . xiii 554
on blood-corpuscles . . xii 570
on the chorda dorsalis . . xii 570
Barry, Sir David, m.d, his death . i 308
his factory report . . xv 295
sketch of his life . . i 611
Barth and Roger, MM., on auscultation . . . . xiii 518
Barthez, M., on diseases of children xii 350
Bartlett, Dr., his peculiar doctrine xx 145
on fever, more remarks on . xviii 180
on his review of typhus . xvii 357
on medical science . . xx 140
on the progress of medicine . xxii 235
on typhus fever . . . xvii 357
Barton, Dr., his treatment of anchylosis . . . . vi 254
on injury of the wrist . . viii 588
Baryta in strumous ophthalmia . ix 552
Barytes, muriate of, in white swellings ii 567
M. Lisfranc on . . . xv 50
Basic unit of Dr. Laycock . . xviii 165
table constituting the . . xviii 166
Basilar artery, peculiar structure of vii 574
Basilisaurus, classification of the . x 211
Basques or Biscayans, origin of the xxiv 464
Bathing and sweating, hydropathic xii 191
in the Cyclopædia of Medicine xxii 434
Bath-men of Germany, account of xiv 404
Bath, alkaline, composition of . i 497
cold, on the tonic effects of . xxii 449
cutaneous absorption in the . vi 334
nitro-muriatic, receipt for . xiii 362
on the nitro-muriatic acid . iv 254
vapour, useful remarks on the xvi 363
warm, caution respecting the . xviii 315
Bath, the waters of . . . xiv 353
Baths, acid, in cutaneous diseases . i 497
how made . . . i 497
alkaline, in cutaneous diseases i 497
Dr. A. Combe on . . i 336
for the poor, Dr. Hodgkin on i 364
in chronic diseases, use of . i 496
in skin diseases, M. Biett on . i 497
in Turkey, account of . . iv 388
of hemlock in skin diseases . vi 538
of iodine for syphilis . . xix 522
of Nero, observations on the . xix 269
of the arms, in croup . . xii 246
public, in London and Brighton v 403
simple tepid, in scaly eruptions i 497
Baths, steam, advantages of . . xix 374
substitutes for the . . i 337
sulphur, in cutaneous diseases i 497
sulphurous, how made . . i 497
tan, mode of forming . . xvii 378
vapour, unsafe for infants . xi 209
warm, use of, in insanity . xxii 58
Batrachian reptiles, the heart of . xii 133
Batters of cotton, unhealthiness of xv 297
Battles, on fevers after . . xi 16
Baudelocque's callipers . . i 106
Baumès, M., his new dermatology xv 372, 399
on venereal diseases . xii 362, 374
Baumgarten, Dr., on plastic surgery xix 396
Baumgartner, his physiology, &c. xxiv 128
Bauer, Mr., his microscopy . xiv 488
Bavaria, medical examinations in . iv 562
Bavarian medical degree, price of a iv 563
universities, account of . iv 562
discipline in . . . iv 563
Bayard, M., on medical jurisprudence xii 174
Baynard, Dr., his use of cold water xxii 432
his pathological reasoning . xxii 433
Baynton's treatment of ulcers, remarks on . . . v 197
Barzellotti, Dr., on medical jurisprudence . . . ix 35, 63
Bazin, Dr., on fibrous membrane of pleura . . . v 220
on nervous systems . . xii 521
Beaufort, Dr., on arsenic . xiii 183, 197
Beaujon, l'Hôpital, account of . i 281
Beau's, M., theory of normal respiration i 480
Beaumont, Dr., case of A. St. Martin ii 375
on digestion . . . v 400, vi 172
Beamuont, Mr., his polypi instruments viii 237
on fistula . . . . . vii 142
Beauty, and sexual attachments . vii 373
Dr. Riofrey and Descartes on v 408
in relation to health . . xv 53
in the human form . . xviii 446
of countenance, Alison on . xviii 445
Sir C. Bell on . . xviii 445
Beccaria's gluten, observations on xvii 15
Beck, Dr., his case of amaurosis . iv 481
obituary of . . . . ix 295
on traumatic hemorrhages . iv 154
his medical jurisprudence . viii 246
his errors on infanticide . iv 99, 100
his researches, &c. . . iv 87
opinion of his essay . . iv 101
Becker, Dr., on temperature of the sea ii 520
Becquerel, M., analysis of urine by xv 505
on temperature of the body . i 246
on urine . . . . xiii 435
on urinary semeiology . xvi 328, 340
Bed-curtains, impropriety of closing v 403
Bedding, airing of, remarks on . i 335
Beddoes, Dr., amusing inconsistency of v 191
on inhalation of ether . . xxiii 549
Bedford, Dr., his translation of Chailly xix 416
Bedford, Mr., on aquo-capsulitis . xvi, 323
Bedlam of Paris, striking scene in . i 286

Bed, new, for fractured neck of femur xvi 486
water, Dr. Arnott's remark on ii 64
Bedrooms, airing of, remarks on . i 335
Beds for patients in Paris, number of i 281
Bedside, physician's duty at the . i 3
Bee, construction of comb of the . xi 97
Beehives, on the nutritive process in xvi 482
Beef-tea, chicken broth, &c. . . xvii 22
Beer, a cause of scandal against . viii 524
considered dietetically . . xvii 22
constituents of . . . xvii 22
Beer, Dr., on epidemic diseases . xix 365
Beger, Dr., on the eye . . iv 480
Behr, Dr. V., his handbook of anatomy xxiii 255
Belfast, statistics of fever in . iii 268
Belgian army, on ophthalmia in . vi 197
towns, mortality in . . iii 50
universities, notice of . . iv 282
Belgium, medical association of . i 606
mortality of towns in . . xix 130
on ophthalmia in . . ix 557
pharmaciens in . . . i 608
statistics of . . . . xix 129
Belknap sewing society in America v 240
Belladonna, as a collyrium, use of . xxi 164
as a preventive of hydrophobia i 25
of scarlatina . xvii 223, xxi 366
as a prophylactic . . . ix 217
clysters in ileus, cases of . iv 220
collyrium of . . . vii 220
epilepsy treated with . . ix 252
harmless to the horse . . iv 130
in diseases of the eye . . x 8
in epilepsy . . . . . xx 261
in incontinence of urine . xxiii 305, 376
in paraphimosis . . . xii 556
in rigidity of os uteri . . xiv 371
in the nose dilates the pupil . ii 263
in typhus fever . . . vi 554
its effects on the brain . . xx 19
its use in epilepsy . . . xx 35
its use in stricture and spasm . ii 261
on the fecula of . . . iii 229
on the use of, in cataract . xi 81
preventive of scarlatina . iv 392
the endermic use of . . v 349
use of, in dysmenorrhea . x 44
use of, in typhus . . . xv 466
Bellingeri, Dr., his researches on
the nerves . . . . ix 141
on Asiatic cholera . . . v 234
on the fifth nerves . . ix 142
Bellingham, criminal case of . xv 87
Bellingham, Dr., his materia medica xiii 521
on compression in aneurism . xx 434
on the hair worm . . v 594
Bellini, Sig., on metastasis . xxiv 288
Bellows-murmur, M. Bouillaud on ii 318
Bellows-sound, experiments on the iii 258
in bronchocele . . . i 486
in endocarditis . . . ii 327-8
in enlarged heart . . . i 483
in meningeal inflammation . i 485
Bellows-sound, mechanism of . iii 257
of the heart . . . . vi 133
on the funic . . . x 274
or bruit de soufflet, its nature i 451
Bell, Dr. C. W., on epidemic ague xvi 558
on an epidemic at Teheran . xviii 369
Bell, Dr. H., on diabetes . . xiv 538
Bell, Dr. J., on regimen and longevity xviii 115
Bell, Mr., on the cure of phthisis. xix 140
Bell, Sir C., Dr. J. Reid on a theory of v 521
faults of his work . . vi 155, 172
his discoveries . ix 96, 106, 121, 140
his grand nervous principle . ix 124
his Institutes of Surgery . . vi 154
his philosophic spirit . . ix 122
his views of nervous system . i 393
Magendie and Mayo . . ix 140
on entrance of air into veins xxiii 447
on expression . . . xviii 442
on fifth and seventh nerves ix 128, 131
on the nerves of motion and
sensation . . . . ii 215
on nervous system . . ix 96
on strabismus . . . xi 492
on the hand, remark on . xxi 116
on the roots of the nerves . xi 278
Bells in Rio, a cause of mortality . ii 234
Belluomini, Dr., on scarlatina xvii 216, 222
Belmas, Dr., on the cure of hernia vi 341
M. his operation for hernia . vi 345
Bene, Dr., his Elementa Medicinæ i 1
Benefit societies, on sickness as to . xxi 15
Benevolent medical fund . . x 294
Bengal, diseases of, Mr. Twining on iii 329
dispensatory . . . . xvi 352, 359
medical returns, Dr. Mouat on xxiii 440
Bengalee misspelling in medical
works . . . . . xxiii 531
Bennet, Dr. J. H., on auscultation xiv 180
his address in Edinburgh . iii 476
on cod-liver oil . . . xiii 197
hydrophobia . . . xii 108
inflammation . . . xix 244
the microscope . . xiv 478, 481
the nervous centres . . xvii 528
the nervous system . . xii 99
Bennett, Dr. E. S., on cherry-laurel water i 263
Bennett, Dr. J. R., on hydrocephalus xv 539
his medical report . . . xx 209
Bennett, J. H., on diseases of uterus xxi 174
Benzoic acid, in calculus and gout . xiii 264
in gout, Dr. Ure on . . xiv 52
use of, in stone and gout . xii 398
Benzoin, fumigation of, in hooping-
cough . . . . . i 574
Benzule, in oil of bitter almonds . xi 134
Berber races of Africa . . . xxiv 442
language of North Africa . xxiv 455
Berg, Dr., on the thrush in
children . . . . . xxiv 422
Beriberi, abdominal affection in . v 127
burning of the feet in . . . v 131
causes of . . . . . v 117

Beriberi, description of . . . . v 118
districts subject to . . v 117
fatality of . . . . . v 117
immediate seat of . . v 121
laryngeal affection in . . v 126
Mr. Malcolmson's essay on . v 116
morbid anatomy of . . v 121
on bloodletting in . . . . v 127
on treatment of . . . . v 127
on various remedies for . . v 128
paralytic symptoms of . . v 119
pathology of . . . . . v 121
state of blood in . . . v 125
state of muscles in 65 cases . v 120
the œdema in . . . v 125
urinary organs in . . . . v 122
Berkshire (U.S.) medical institution ii 589
Berlin, bad lunatic asylum at . i 500
clinical observations in . xxiv 41
delirium tremens in . . xiv 561
epidemic constitution of, 1837 vi 220
lying-in hospital, report of . v 576
medical statistics of . xxi 205, 213
surgical pharmaceutical studium in i 298
university, account of . . i 289
veterinary school . . vi 513
Bermuda, climate of . . . . xii 172
consumption prevalent in . viii 222
Berndt, Dr., his clinical communications . . . . . . ii 176
Bernt, Prof., his test of respiration ix 47
Bernouilli, his physiological opinions i 384
his theory of muscular action i 384
Berres, Dr., on the nervous substance . . . . . vi 394, 399
on the structure of nerves . v 219
Berton, Dr., on diseases of children xv 320
Bertrand, M., on animal magnetism . . . . . xix 428
Bethlem, class above paupers in . xx 478
amusements in . . . xx 474
former bad state of . . ix 146
great improvements in . . xx 472
management of . . . xiii 215
on pupils at . . . xiv 217, xx 477
reports of . . . . . xx 471
statistics of . . . . . xviii 371
Bethnal-green, longevity in, rate of xv 306
Bezoar of the deer, medical effects of xi 233
stone, its reputation in Turkey iv 392
Bhotiyahs inhabiting Thibet . . xxiv 462
Bibliography, medical, by Dr. Forbes i 513
Bibra, Dr. v., his zoochemical tables xxiv 286
Biceps humeri, displaced tendon of xiv 63
Bicêtre, at Paris, account of . . i 281
Dr. Conolly's account of . xix 292
improvement in the . . xix 297
schools in the . . . xix 295
striking scene in . . i 286
Bichat, admitted fluid vitality . iii 385
a partial follower of Stahl . iii 384
commendation of his works . iii 386
difficulty in his system . iii 385
Bichat, his nosological opinions . iii 385
his opinion on fevers . . iii 386
his pathological opinions . iii 385
his physiological principles . iii 385
his system of anatomy . . xiv 487
on his general anatomy . iii 384
Bichat's researches, their effect on physiology . . . i 388
Bichel, a German murderer, case of xxiv 235
Bichloride of mercury, Biett's pills of xxiii 365
Bichromate of potash, poisoning by xviii 552
Bickerings, professional, remedy for ix 429
Bicorn malformation of the uterus . xv 104
Bidder, Dr., his neurological researches vi 204
on the sympathetic nerves . xvii 379
Bidloo, the favorite of Peter the Great i 604
Bierkowski, Dr., his Polish report iii 475
Biett, M., his lotion for scald-head i 578
on syphilitic eruptions . xxiii 345
on the use of baths . . i 497
Bièvre stream in Paris, effects from xi 18
Bigelow, Dr., on ether in operations xxiii 310
on pneumothorax . . . vii 569
on self-limited diseases . iii 200
Biggar, a dangerous disease of India iii 353
Bigger, Dr., on transplanting the cornea iv 537
Bigsby, Dr., on colica pictonum . vii 284
Bile, absence of, in fæces, Liebig on xiv 506
and urine, waste of system in . xiv 514
agents affecting . . . xxi 42
Berzelius's analysis of . . xvii 434
carbon supplied by . . xiv 303
chemico-physiology of . . xiv 514
composition and properties of xxi 40
constituents of . . . xii 280
dilution of chyme by . . xxiv 277
duct, structure of . . . xii 133
ducts, M. Krause on . . xxi 559
empirical formula of . . xiv 514
healthy and calculous, analysis of vi 247
in blood, effects of . . iii 327
experiment on . . vi 446
observations on . . xvii 435
its action on chyme . . xvi 223
its influence on digestion . xxi 563
non-elimination of . . xii 280
observations of Scherer on . xxi 561
on deficient secretion of . xxi 56
on excessive secretion of . xxi 56
Liebig on the nature of . xiv 303, 308
on the uses of . . . xvii 435
reabsorption of, fact against . xvii 353
secretion and properties of . xix 276
solution of blood-corpuscles by xvi 147-8
casein by . . . xvi 148
sources and uses of . . xxi 41
unhealthy state of . . . xxi 57
Biliary calculi, composition of . xvii 447
jaundice, &c. . . . xx 244
varieties of . . . xvii 446
calculus, case of a large . . xviii 366
diseases in animals . . xxiv 84
duct, common occlusion of . xxi 54

Biliary ducts, case of dilatation of . v 115
inflammation of . . xxi 52
Bilious and typhoid fever, identity of viii 433
dyscrasy, characteristics of . xxii 312
Dr. Horaczek on . . xxii 311
treatment of . . . xxii 317
disorders, dialogue on . . vii 476
on purgatives in . vii 477, 480
treatment of . . . vii 481
use of bathing in . . vii 481
fever, case of . . . viii 433
post-mortem appearances in viii 434
or choleric temperament . xv 424
Billard, Dr., on diseases of infants xi 198, 209
Billing's, Dr., principles of medicine v 200
Billion, time required for counting a xi 138
Bill, new medical, alterations in . xix 549
of health, nature of a . . xvi 301
Bills of health, the different kinds of xvi 302
Bilocular malformation of uterus . xv 104
Bimeconate of morphia, Dr. Thomson on . . . . viii 276
Mr. Squire on . . . viii 275
Binders, obstetric, figures of . . vi 568
Bingham, Mr., on the insane . xii 54
Bini, Dr., on clinical medicine . xxiv 360
Biniodide of mercury, ointment of . . . xix 518, xxiii 370
preparation of . . . xiii 76
qualities of . . . . xiii 75
Binns, Dr., a lucus à non lucendo by xv 185
his anatomy of sleep . xv 184, 187
Biographical dictionary, new xiv 536, xix 530
Biographies, English, puerilities in . iv 76
Biography, ancient medical . . xv 106
medical . . . . . xix 533
observations on . . . xix 530
Biospheres, Dr. Mayer's romance of the iii 467
Biotic forces, Unzer on . . . xxiv 182
with consciousness . . xxiv 184
Bird, Dr. Golding, his Natural Philosophy . . ix 529, xvii 523
on a new test of pregnancy . xii 326
on charcoal vapours . . ix 377
on diseases of children . . xxiii 424
on electricity as a remedy . xii 344
on Liebig's theory . . xv 504
on urinary deposits xvi 312, xx 58, xxiii 248
globules . . . xvi 321
Bird, tubercles in the air-cells of a . iv 244
Birds, brain of, error of Mr. Noble on xxii 501
cerebellum of . . . xxii 539
diffusion of . . . . iii 369
highest bodily heat in . . xii 148
lymph-globules of . . . xiv 571
peculiarities of the brain of . viii 228
structure of the iris in . . v 553
the little drinking of . . xvii 348
Birkett, Mr., his Behr's anatomy xxiii 255
Birmingham Dispensary, reports from vi 391
Infirmary, reports from . . vi 391
low ratio of phthisis in . . vi 23
medical school of . i 207, vii 520, 522
Birmingham reports, by Mr. Ryland ix 222
traffic in children by . . xiv 457
Birnbaum, Dr., on the cervix uteri xvi 184
Birth, accidental death of child at . vii 139
live, signs of . . . . xvi 310
of living child on 179th day . xiv 256
of man, aphorisms on . . xix 557
plurality of, cases of . . xxiii 280
respiration of child during . iv 101
what constitutes it, in law . ix 44
Births and deaths, diurnal periods of xxiv 114
in England . . . xi 422
on registration of . . xviii 205
at lunar phases, table of . xviii 177
deaths, and marriages in England xxi 2
in other countries . . xxi 2
on precocious and retarded . ii 403
plurality of, more dangerous than single . . . ii 90
Bischoff, on maturation of the ovum xix 84
on medicine and surgery . xvi 507
on oval development . . xvi 513
on transfusion of blood . . vii 548
Bishop, Mr., on the human voice . ii 214
Bismuth, oxide of, in yellow fever . iii 217
Bitters, medicinal action of . . xiv 144
Bizot, M., his researches on the heart vi 41
Black, Dr., his retrospective address xv 228
tranquil death of . . . vi 497
Black bodies on the cornea, nature of ii 269
Blackburn, Mr., on the articulations x 330
on diseases of joints . . xiii 169
Black death and cholera, Mr. Parkin on xiii 498
discoloration, Dr. Carswell on i 136
expectoration, Dr. Thomson on . . . . iv 150, vii 154
lion of Portugal . . . x 407
lungs, anatomy of . . . iv 150
matter of lungs, Dr. Christison on i 135
Blackening of bronchial glands . i 137
Blackmore, Dr., on nervous diseases xxi 273
Black-snake root, use of, in chorea x 280
Black troops, fatality of phthisis in vi 150
in Africa, mortality in . vi 150
vomit in yellow fever . . x 278
Blackwood's Magazine, mesmerism in xix 485
Bladder, abscess in walls of . . xiii 237
and perineum, Dr. Monro on . xiv 540
and prostate, diseases of . x 247
and urethra, on neuralgia of . iii 231
and uterus, prolapsus of . xiii 563
cancer of . . . . . xxii 305
case of earpick in . . v 253
catarrh of, injections in . iii 521
catheterizing the female . iii 423
chronic inflammation of vi 509, 565, xi 360
Civiale's mode of injecting . vii 167
congenital inversion of . vii 414
croupous inflammation of . xv 103
cure of fistula of . . vii 414
different inflammations of . vi 510
diseased, Sir B. Brodie's case of xii 122
diseases of, Mr. Coulson on . xv 228

Bladder, distended, mistaken for inflammation . . . xiv 370

distension of, percussion in . i 487

Dr. Prout on morbid growths in xi 361

extrophy of . . . . . xiv 573

fungus of, diagnosis of . . xi 362

irritable, Dr. Prout's account of xi 360

with diseased kidney . xiv 162

with renal disease, treatment of . . . xi 362

irritability of . . . vi 509

Mr. Coulson, on diseases of the vi 509

nervous irritability of . . xi 362

paralysis of, cantharides in . ii 549

treatment of . . viii 280

pock in the cow . . . ix 95

polypous excrescences of . xi 361

prolapsed, case of . . . xxii 319

puncture of, researches on . xiii 241

puncturing the . . . v 467

soot in diseases of . . v 575

tubercle in . . . . . xv 103

typhous process on . . xv 103

wounds of the . . . . ix 61

Blake, Dr., on delirium tremens . x 550

Blake, Mr., on injections into the veins vi 577

on the action of poisons . ix 569

on the blood . . . xii 571

Blakiston, Dr., on influenza v 214, vii 95

Blanche, Dr., on severities to lunatics . . , xi 104, 125

Bland, Dr., his pills for chlorosis . viii 252

improvement of his pills . viii 252

Blandin, M., on amputation of the foot vi 535

on erysipelas . . . vii 252

on plastic surgery . vii 386, 416

Blane, Sir G., on hydrocephalus . ix 231

on instinctive actions . . v 529

Blankenheimer thee, receipt for . xx 449

Blasius, Dr., on amputation . viii 205

on aneurism . . . viii 413

plate of his amputating knife . viii 206

Blastemal formations and cells . xxi 422

Blastoderma, area germinativa in . xvi 547

cumulus germinativus in . xvi 547

formation of the . . . xvi 546

layers of the . . . ix 17

or germ-membrane . . ix 17

Blatin, M., on female discharges xiv 534

Bleeding, effects of in inflammation viii 199

from hemorrhoidal veins . viii 497

M. Bouillaud's new formula of vi 367

in abdominal inflammations . xiv 5

in apoplexy, remarks on . xx 198

in inflammation of brain . xiv 5

injudicious, caution on . xxi 370

in old age, weakening effect of vi 49

in pneumonia, effects of . xx 80

in traumatic inflammations . xiv 5

its effects in rheumatism . xx 185

local, when applicable . xiv 5

repeated, mercury a substitute for i 399

topical, rationale of action of . viii 199

Bleeding, to syncope, Dr. Wardrop on ii 174

Blennophthalmic matter, contagion of xiii 239

Blenorrhœa, popular cure for in Paris xii 50

Blenorrhagia, abortive treatment of xii 370

and blennorrhœa, the terms . xii 363

causes of . . . . . xii 365

in females, pathology of . xii 365

rife in Paris . . . xii 365

in women, cure of . . i 271

Mr. Acton's theory of . . xii 363

syphilides from . . . xxiii 363

Blepharoplastic operations vii 404, xxi 308

Blepharoplegia, use of galvanism in v 34

Blepharoptosis, operation for . xxi 309

Blind, &c., effect of civilization on . xviii 241

persons born, phenomena in . xxiv 372

Blindness cured, interesting case of xii 567

from slight injuries . . ii 268

use of oxygen gas in . . xix 234

Blister, magistral, of M. Valleix . xviii 289

on knee, fatal phagedæna from ii 15

Blisters, applied over the eye . iii 450

Dr. Spillan's remarks on . iv 113

exaggerated utility of . . xii 34

in abdominal inflammations . xii 34

in pleurisy, doubts on . . ix 317

in puerperal fever . . . ii 92

in sciatica . . . . . i 499

M. Louis' remarks on . i 413, vii 56

observations on . . . xvi 205

the use of . . . xiii 64

on their use in children . . xiii 65

over the eye in ophthalmia . iii 513

Blizard, Mr., biographical sketch of vi 288

Blizard, Sir Wm., memoir of, by Mr. Cooke . . . . . ii 205

Blondlot, Dr., on digestion . xviii 529

Blood, absorption of oxygen by . viii 270

abstraction of fibrin from . viii 330

action of ammoniacal salts on xvi 153

lymphatic glands on . xvi 226

medicines on . . . viii 355

on nervous system . . xxi 146

pus on . . . . xvii 148

after death, Dr. Davy on . viii 272

table of state of . . viii 272

albumen of the . . . viii 348

in the globules of . . vi 440

analysing, on processes for . xvii 430

analysis of . . . . ii 281

L'Heritier on . . . xvii 429

globules of . . vi 439, 443

in chlorosis . . . vi 447

anatomy, physiology, and pathology of, Mr. Jones on . xiv 585

and lymph, and flesh-juice, salts in xxiv 269

and nerves, Dr. Heidler on . xxii 218

and other fluids, experiments on xii 528

Andral on the pathology of . xvii 136

and respiration, relations of . vii 279

and urine, analysis of . . xxii 223

animalcules in serum of . . viii 351

arterial and venous, difference of vi 444

Blood, ashes of, analysis of . . xix 254
behind uterus, case of . . xviii 89
bile in, experiment on . . vi 446
buffy coat of . . . . viii 347
formation of . . xiv 589, xvii 250
microscopy of . . xxiii 507
in inflammation . . xvii 147
in pregnancy . . . xvii 148
on chlorotic . . . xvii 141
carbonic acid of . . . i 590
gas in . . . viii 274
catamenial, acidity of . . ii 274
changes in chemical composition of xxii 327
in inflammation . . xviii 255
in stagnant . . . xviii 255
in the capillaries . . viii 175
in typhus . . . viii 443
of colour in . . . xix 253
of the, in Bright's disease xvii 151
of, in inflammation . xvii 144
chemical composition of . . xix 254
chemistry of the . . . xvii 254
Dr. Griffith on . . xxiii 427
circulation of, in capillaries xiv 599
in fishes . . . . viii 171
in insects . . . ii 213
coagulation of v 102, xii 440, xiv 588
after death . . . xi 255
Dr. Polli on . . . xvii 249
in vessels of brain . . xvii 487
remarks on . . . viii 180
report on . . . xxi 543
collection of oxygen gas from . xvi 211
colour of, in various diseases . xxii 327
colouring matter of . . vi 435, 438
colourless corpuscles of . . xiv 588
relations of . . . xiv 598
components of, properties of . xvii 428
composition of . . . xvi 210
of the portal . . . xvi 214
condition of, in Bright's disease xix 32
in pneumonia . . . xx 76
in dropsy . . . xvii 150
constituent parts of . . vi 432, 434
constituents of, in plants . xiv 302
diseases of . . . xvii 480
copper in . . . . . vi 433
course of, when retrograde . vi 135
creamy appearance of . . vi 448
cruor of, after death . . viii 274
culture of the renewal of . xvi 224
death beginning with the . xvii 501
from loss of . . . v 175
depuration or moulting of . xvi 214
determination of . . viii 181, 515
Dr. Williams on . . xvii 495
to the head . . . xvii 200
diabetic, analysis of . . vii 457
on the sugar in . . xviii 369
dilution of, effects of . . viii 320
diseased, alleged cause of insanity xvii 526
saline medicines in . iii 263
diseases from changes in . vi 448
Blood, Dr. Davy's experiments on . viii 270
Dr. G. Burrows on vitality of . viii 180
Dr. G. O. Rees on . . . xxi 544
Dr. Mandl on . . . xi 225
Dr. M. Barry on, dissent from . xiv 586
Dr. Wilson's enthusiasm on . xvi 424
effects of alkalies on viii 345, 355, 356
of carbonate of soda on viii 342
of certain salts on . . xvi 149
of loss of, on the vessels . iii 221
of respiration on . . xvii 484
of sulphuric acid on . viii 345
effused on brain, absorption of iii 321
elements of, in the excretions . xiv 307
empirical formula of . . xiv 514
examination of after death . viii 273
excess of, spleen a reservoir for i 565
exhaustion from loss of, effect of xiv 57
experiments on formation of . xvi 224
febrile, reduction of solids in . xvii 143
fever, characteristics of . viii 441
Dr. Kehrer on . viii 428, 440
fibrin in . . . vi 439, 440
floating fibres in the, Mayer on xiv 295
gas extracted from, by air-pump viii 271
gelatin in the . . . vi 433
general constitution of . . xvii 249
granula in buffy coat of . . xiv 553
great losses of, in diseases . xxi 308
gray granulations in the . vi 135
Harvey's views of life of . i 388
healthy, quantity of fibrin in . xviii 258
heat in relation to state of . xii 147
heat of arterial and venous . xviii 520
hemorrhoidal, nature of . . xvi 227
Hewson on, notice of . . v 101
Hunter on life of, remarks on . i 388
Hunter, J. on physiology of . v 48
hydatids free in . . . xx 412
hyperinosis of . . . xviii 257
hypinosis of . . . xviii 257
imperfect aeration of . . vii 264
impoverished, diseases from . viii 324
in animals, variations in . xvi 225
in capillaries, circulation of . xiv 575
in cholera, analysis of . . vi 447
in diabetes, on sugar in . . vi 446
in fevers, M. Andral on . . xvii 143
inflammatory, on changes in . xx 78
source of fibrin in . . xviii 260
in health, state of . . . xvii 567
in hemorrhage, M. Andral on xvii 149
injection of foreign substances into viii 330
in inflammatory affections . vi 446
in jaundice, state of . . vi 446
inorganic compounds in . xii 571
in pregnancy, M. Andral on . xvii 148
in scurvy, on condition of . xx 152
instrument for measuring force of viii 336
in the head, Dr. Kellie on . iii 323
in the heart, Dr. Wardrop on ii 218
in winter-sleep . . . xxiv 409
its changes in respiration . viii 480

Blood, its state, in Bright's disease . xviii 336

in fever . . . . xx 347

life of the, Hunter on . . viii 178

loss of, Dr. Nasse on . . iii 221

often fatal in erysipelas . ii 26, 27

lost, females easily repair . xiv 5

M. Denis on the chemistry of . vi 431

menstrual, injurious . . ix 545

microscopic characters of x 257

nature of . . . xvi 228

M. Lecanu on the . . . vi 431

M. l'Heritier on the . . xvii 427

M. Magendie on the . . viii 341

mode of preventing the flow of v 574

morbid, chemical analysis of . xii 270

matter in, counteracting . xvii 486

matter in, expelling . xvii 486

quantity of fibrin in . xviii 258

motion of, in capillaries viii 175, xviii 102

movement of, in veins . . viii 171

moving forces of the . . viii 169

powers of . . . xix 557

muscular action in relation to xvi 421

new analysis of the . . vi 248

new pathology of the . . xvi 227

occurrence of urea in the . xiii 228

office of the . . . . xviii 101

of cholera, inoculation with . ix 245

of man and animals different . xvii 136

of the capillary system . vi 445

organizability of coagula of . viii 181

passage of, through its vessels viii 339

pathology of . . . iii 66

pathological analysis of . xx 76

changes in . . . vi 445

relations of . . . xxii 326

peculiar milky appearance of . xvii 433

plasticity of, in inflammation . xx 71

poisons to be found in . . xx 320

powers which move . . xvii 490

predisposing causes of disease in xx 341

presence of iron in . . vi 434

of urea in . . . vi 433

pressure of, in arteries . . viii 337

in veins . . . . viii 337

preventives of perfection of . xvi 226

products from . . . xviii 102

progress of knowledge of . xix 252

promoters of aeration of . vii 265

proportion of albumen in . vi 444

of fibrin in . . . xvii 136

of globules in . vi 439, xvii 137

of serum in . . . vi 439

of solids in . . . xvii 137

pus-like globules in . . xv 234

quantity of red corpuscles in . xviii 258

within skull, not fixed . xxii 408

Rasori on buffy coat of . viii 202

reaction after loss of, nature of iii 326

red globules of, L'Heritier on . xvii 432

red particles of, disorders of . xvii 481

remark on poisons in the . vi 448

reorganization of . . . xviii 277

Blood, report on the . . . xvii 249

researches on, by Gmelin, &c. i 590

ripening of . . . . xvi 226

salts in the, derivation of . xvi 149

self-moving power of . . iii 258

singular appearance in the . v 97

solution of fibrin in . . vi 442

specific odour of the . . xi 226

stagnant, Dr. Hastings on . xviii 255

stagnation of, in capillaries . xvii 493

stains, examination of . . xvi 74

state of, in disease of heart . vi 447

after loss of the kidneys . i 592

in congestion . . viii 182

in jaundice . . . xvii 432

in marshy districts . . xxi 211

in starved dogs . . xvii 145

in typhus . . . vi 447

in various diseases . . xi 242

substances preventing coagulation of viii 345

which coagulate . . viii 345

which liquefy the . . viii 345

the, in mercurial stomatitis . xvii 145

theory of inflammatory . xx 77

the seat of morbid poisons . xvii 485

torpid, stagnation of . . iii 211

transfusion of . . . vii 548

typhoid, appearances in . . viii 442

venous analysis of . . vi 443

morbid relations of . xx 430

viscosity of . . . . viii 323

vital changes of, in the vessels viii 180

vitiated, nervous disorder from iii 326

worms in . . . . v 555

Blood-corpuscles, Blood-globules,

Blood-vesicles . . . xi 448

are true animal leaves . xvi 227

as carriers of oxygen . xiv 596

as glandular cells . . xiv 597

as maintaining excitability xiv 597

attractions and repulsions of xiv 599

cells or vesicles . . ix 509

changes of . . . xvi 210

colourless . . . xvii 93

in capillaries . . xiv 598

composition of . . xiv 262

and properties of . xvi 210

conversion of, into pus . viii 193

development and death of xvi 212

dissolved by bile . . xvi 147

Dr. Barry on . . xii 570

elasticity of . . . vi 220

experiments on the . . xvi 211

floating gland-cells . . xvii 251

form of . . . . xiv 585

and size of . . xiv 260

function of the . . xvi 213

in relation to nutrition . xiv 600

in sputa . . . xvii 520

in the chick . . . xvii 251

Magendie on . . . viii 351

motion of, in vessels . viii 339

motions of . . . vi 219

Blood-corpuscles, Blood-globules, Blood-vesicles moulting of . . . xvi 213

Mr. Addison on . . xviii 92

Mr. Gulliver on . . xxi 543

nature and uses of . . xiv 59

nucleus of . . . xiv 586

of the camelidæ . . xvi 287

of the Stanley musk deer xvi 287

of vertebrata, oval . . xiv 570

on diseases from . . xvi 213

origin of xiv 223, xv 275, xvi 212

physiology of . . . xiv 595

production of . . . xiii 229

red, use of . . . xv 279

report on . . . xvii 250

secretion by . . . xiv 597

size of . . . . xiv 585

solvents of . . . viii 355

structure of . xiv 133, 261, 585

the smallest . . . xiv 570

white, use of . . . xv 278

Blood-diseases, Dr. Todd on . . xvi 462

Blood-horse, Mr. Knight on . . vii 381

buffy coat in, normal . xvii 136

Bloodletting, an injurious effect of . xvii 483

as a diagnosis, Dr. Hall on i 204

awful impiety of . . xv 127

by physicians of Padua . xxiv 360

copious, at Turin . . ix 225

Dr. Clutterbuck on . v 188

Dr. Marx on . . . xvi 475

Dr. Paine's philosophy of xi 390

Dr. Symonds on . . xxiii 602

Dr. Wardrop on . . ii 171

English, opinion of, in Paris ii 63

false doctrines on . . xviii 72

false indications for . vi 516

general, in hydrocephalus i 20

in ague, Dr. Mackintosh on iii 104

in cold stage of agues . xvi 563

indiscretion in . . v 387

in fever, history of . . i 44

in fever, remarks on ii 63, xxiv 214

in inflammation . . xviii 72

in intermittents . . viii 64

in mania . . . xiii 559

in old people . . xiv 5

in ophthalmic diseases . xi 55

in ophthalmia, remarks on ii 347

in pneumonia . . . xiii 402

Dr. Jackson on . iii 456

table of . . . i 405

in puerperal fever . . ii 92

in rigidity of os uteri . ii 79

in the arm, Lisfranc on . xiv 21

in uterine affections . xiv 6

in yellow fever, Dr. Bone on xxiii 428

its effect on particular symptoms of pneumonia . i 410

mischief of injudicious . vi 516

M. Lisfranc on . . xiv 5

on the abuse of . . vi 515

Bloodletting, M. Louis on . . i 397, 405

on the section in . . vi 229

relief of dyspnœa by . i 410

renovating influence of . xxiii 580

revulsive effect of . . xiv 5

remarks on . . i 496

tolerance of, Dr. Hall on i 204

Blood-vessels, absorption of . . xvii 260

and muscles, relation of . x 354

carcinoma in . . . i 129

Dr. Sharpey's experiment on xviii 490

effect of loss of blood on iii 221

Mr. Crisp on diseases of . xxiv 410

Mr. Liston on injuries of . xxii 372

muscular fibres of . . x 551

on absorption by . . xix 280

on contractility of the . x 551

on deviations of . . xix 487

on the vital action of . iii 16

Bloody tumours of the scalp in infants i 182

urine, observations on . . xv 478

Bloomingdale lunatic asylum . vii 153, ix 195

Blosfeld, Dr., on forms of lepra . iv 227

Blows, case of death from violent . iii 248

Bloxam, Mr., on the placenta . xi 459

Blue diseases of infants . . xi 208

pill, alleged poisoning by . xviii 543

Bluff, Dr., obituary of . . vii 298

Blumenbach, his museum at Göttingen i 500

his pocket writing . . . xv 26

Blushing, cause of, explained . . v 202

observations on . . . xii 128

physiology and mechanism of viii 245

Boa constrictor, spinal cord of . viii 227

spinal nerves of . . vii 227

Boddington, Mr., on consumption x 549

Bodenius, Dr., on scarlatina . xvii 216, 224

Body and mind, reciprocal influence of xv 413

and soul, Mr. Redford on . xxiv 287

chemical composition of the . xxi 541

morbid habits of . . . xxiii 59

rudimentary formation of the . viii 9

Boeck, Dr., on Norwegian leprosy . xviii 346

Boehm, Dr., on structure of the intestinal glands . . . i 521

Boerhaave, his idea on smallpox . vi 483

his physiology . . . i 386

Boiled and roasted meat, tenderness of xxiv 271

Boiling, effects of, on meat and vegetables . . . . ii 282

meat, Liebig on . . . xxiv 271

of food, observations on . . xvii 36

water, an effect of swallowing xi 417

in callous fistulæ . . xi 252

swallowing . . . v 439

Boils and buboes, iodine in . . viii 521

and eruptions in hydropathy . xii 192

on the seat of . . . x 349

the sloughs or cores of . . x 350

Boismont, M. de, on menstruation xiv 382, 396

Boisragon, Dr., on osteology . vii 537

Boisseau, Dr. biographical sketch of iii 297
obituary of . . . . . iii 297
Boivin, Mme. and Duges, on uterine diseases . . . ii 517
Boleyn, Anne, mammæ and fingers of xxii 156
Bolting food in America, remarks on xix 27
Bolton, Dr., on the iris . . viii 580
Bologna, medical school at . . i 498
society, memoirs of the . . v 167
Bombastic, origin of the word . xiv 148
Bombay, diseases of, Dr. Morehead on xxi 381
medical society of, Trans. of . xiii 347
medical transactions of . . xxi 373
Bona fever, case of . . . viii 58
treatment of . . . viii 58
statistics of fever at . . viii 57
Bone, Dr., on yellow fever . . xxiii 426
Bonaparte, his opinion of medicine ix 478
Bone, a definition of, Prof. Owen on xxiii 476
and cartilage, structure of . i 231
atrophy of, Mr. Curling on . iv 152
blood-vessels of, compact . xiv 269
bruises of, serious effects of . xv 51
canaliculi and lacunæ of . xxiv 389
chemical composition of . xvii 261
curious case of absorption of . v 244
deposition of fat on . . xxiv 397
development of . . . xix 589
fibrous structure of . . xxiv 388
formation of, conclusions on . xxiv 396
by periosteum . xxiv 395, 401
granular structure of . . xxiv 387
growth of, J. Hunter on . xv 204
M. Flourens's summary on xxiv 405
superposition in . . xxiv 400
inflammation of . . . ii 536
M. Richet on . . xxi 128
inner layers of, absorption of . xxiv 403
lacunæ of, deposit between . xxiv 393
laminæ of, excavations in . xxiv 389
minute structure of . ix 513, xiv 268
Müller on formation of . . viii 286
on growth of . . v 79
nature of caries of . . ii 536
not necessary to the skeleton . xxiii 476
on absorption of dead . . vii 140
on madder-experiments on . xxiv 406
on morbid anatomy of . . ii 535
on necrosis of . . . xii 157
on pulsating tumours of . xxiii 412
on structure of . . . ii 535
and economy of . . xx 305
on ulceration of . . . xx 292
partly destroyed by fire, plate of i 326
power of periosteum to form new viii 285
primary areolæ of . . . xxiv 392
regeneration of entire shaft of xii 157
M. Textor on . . xviii 529
Bone-setters of Turkey, their success iv 396
in Switzerland, account of vi 312
Bone-setting family in the Vosges vi 312
Bone-soup in France, account of . xiii 494
Bone, softening of, cause of . . xii 158
pathology of . . . xx 100
plate of . . . . i 325
structure of, report on . . xvii 262
treatise on formation of . . xxiv 386
Bones, action of madder on . . x 257
after periods of interment . xvi 71
analysis of earthy matter in . xxiii 425
in mollities . . . ix 389
and joints, treatises on diseases of xxi 124
as a manure, Liebig on . . xvii 226
buried, alterations in . . xvii 262
caries of . . . . . xii 156
chemical analysis of . . xxi 547
composition of . . . xvii 444
Cuvier on the number of . xxiii 478
diseases incident to . . xii 159
from injuries to . . vii 148
distinguishing the sex of . xvi 71
Dr. Medici on structure of . i 549
earthy matter in . . . vii 154
excision of ends of . . xi 459
existence of fluorine in . . xix 257
exostosis of . . . . . xii 159
experiments on, by Medici . i 550
fibrous structure of . . i 550
flexion of long . . . xiv 7
Haversian canals of . . xiv 269
hereditary brittleness of . xxii 49
inflammation of . . . xii 155
influence of madder on . . ix 272
long, fractures of, Mr. Liston on xxii 369
free communication in . xxi 129
increase of girth of . xxiv 395
morbid enlargement of . . xxi 134
growths in and upon . xii 159
Mr. South's description of the iii 491
new episternal, discovery of . viii 248
of the aged, on fragility of . xx 117
organic diseases of the . xii 154
partial fracture of . . . xiv 7
pathology of . . . vii 566
regeneration of . . . xvi 504
reparation of . . . . iv 367
resection of, tetanus after . xx 51
Scarpa's opinion on, disputed i 549
simple and compound . . xxiii 479
structure and development of xxi 547, 548
suppuration in . . . xii 156
syphilitic affections of . . x 416
writers on diseases of the . xii 154
Bonetti, M., on arteritis . . viii 556
Bonetus, object of his morbid anatomy vi 296
Bonino, M., on resection of head of femur . . . . . xxi 124
Bonnet, M., his mode of curing hernia vi 345
on diseases of joints . xxi 124, xxii 62
liver . . . . . xii 78
on extirpation of the eye . xv 244
on extraction of cataract . xv 245
on the treatment of varix . ix 252
Bonn university, account of . i 292
Book-clubs, injurious to large journals xxiv 592

Books, apologies for imperfections of xiii 136
on medico-popular . . xix 234
on writers of medical . . iii 355
procrastination of promised . xxi 499
theory of the cause of bad . viii 542
Booth, Rev. Dr., on education . xxiv 284
Boott, Dr., his life of Dr. Armstrong i 34, 67
Borax, on its use in labour . . xii 479
use of with ergot of rye . vi 86
Border medical society . . ix 542
Borelli, his great attainments . i 383
his physiological principles . i 383
Bosch, M., on the Indian thrush . xii 516
Bosjesmen of South Africa, account of xxiv 76
Bostock, Dr., analysis of calcareous
tumours by . . . ii 164
his system of physiology . iii 477
his work on physiology . . xiii 467
on examining urine . . vii 142
on typhus fever . . . xv 151
Boston Medical and Surgical Journal vi 260
(U. S.), Mr. Combe on the heads in xv 57
Botanical nosology, account of a . iii 452
prize medal . . . iii 293
Society of Edinburgh . . ii 611
Botanists, cause of suicide in . v 374
unchemical researches of . xi 439
Botany and zoology, new magazine of ii 301
British, Dr. Macreight on . vi 511
Dr. Lindley's synopsis of . vi 511
Dr. Willshire's principles of . x 248
Himalayan, Mr. Royle on . iii 205
in relation to medical studies x 187
manual of British . . . xvii 248
Mr. Lindley's school . . viii 538
of the Himalayan mountains . i 215
structural and physiological . xxiv 223
various works on . . . iv 1
Botheratio-therium, story of . . x 211
Bothrycephalus, account of . . xx 418
Bouchardat, M., his gluten bread xvi 484
on fibrin . . . . . xv 229
Bouchut, M., on diseases of infants xx 357
Boudin, M., on marsh fevers . xiv 43
on phthisis and typhoid fever . xxii 211
Bouillaud, Dr., his work criticised iii 391
his retort to M. Thiers . . xxiv 262
his treatise on the heart . ii 309
on air in the veins . . vi 517
on diseases of the heart . . i 424
on glanders in man . . xiii 538
on medical philosophy . . iii 384
on rheumatism . . . vi 352
on sanguineous concretions . ix 549
Parisian laudits of . . . xiii 405
Bougard, Dr., on delirium tremens xvi 506
Bougie, fucus esculentus, use of, as a ix 222
Bougies, antiquity of . . . xiii 316
caustic, mode of using . . xiii 321
cautions on the use of . . v 467
cure of dysmenorrhœa by . iii 113
false passages by . . . xiii 323
for dysmenorrhœa, Dr. Simpson's xx 539
Bougies, ivory . . . . . viii 258
made of elm bark . . . vi 259
of Heliodorus . . . xiii 316
of Van Gesscher . . . xiii 317
pliable, objected to . . v 467
rectum, warning case as to . x 45
with caustic potash . xiii 320, 324
Bouquet of wines . . . . xvii 22
peculiar, of wine, cause of . viii 330
Bourbon, isle of, in relation to cholera xxiii 330
mortality of slaves in . . vi 150
Bourgery and Pancoast . . xx 39
M., on the lungs . . . xiv 546
on the spleen . . . xiv 541
Boussingault and Dumas on organic chemistry . . . xviii 531
M., on the food of animals . xxiii 157
Bouvier, M., on section of tendo
Achillis . . iii 233, viii 385, 405
Bowdich, Dr., his translation of Louis v 549
his young stethoscopist . . xxii 209
Bowman, Mr., on the Malpighian bodies xiv 567
Bow, Dr., on the external use of opium iv 243
Bowel complaints in army and navy xv 16
in Malta . . . ix 67
in West Indies . . ix 66
wounded, action of ligatures on xxiii 14
Bowels, diseases of, in children . xvii 560
hemorrhage from, remark on . vi 139
obstruction of, diagnosis in . xxiii 419
treatment of . . . xxiii 420
on daily evacuation of . . vii 477
treatment of obstruction of . viii 496
Bowerbank, Mr., on shells . . xxi 62, 81
on structure of shells . . xx 376
Bowman and Todd, their anatomy, &c. xv 537
their physiology . . xx 201
Mr., on fatty liver . . . xiii 553
Bowring, Dr., on the plague . vi 582
on plague and quarantines . viii 233
Boyd, Dr., on malformation of
duodenum . . . . xxiii 412
Boyer, Baron, his treatise on syphilis v 2, 29
Boy, intractable, interesting case of a xxiv 233
Boyle, Mr., on the coast of Africa xi 364, 382
Brachet, his experiments on hunger ii 370
on hypochondriasis . . xx 364
on intermittent fever . . iiii 105
Brachial artery, case of obliteration of iii 242
deviations of . . . xix 499
ligature of . . . xix 501
on tying the . . . v 463
plexus, concussion of . . xv 401
Brachycephalæ, or short heads . xviii 373
Bradyfibrine and parafibrine . . xxi 543
Brady, Mr., his translation of Fournet xiii 220
Braid, Mr., his neurypnology . xix 428
his hypnotic process . xix 476
Brain, abnormal growth of the . vii 223
absorption of blood effused on iii 321
abscess of the . . . xx 229
absurd names of parts of the . iv 487
acephalocysts in the . . xi 524

Brain, action of, without consciousness xxiv 204

material ideas on . . xxiv 192

acute softening of, duration of xvi 14

gray . . . . xvi 4

invasion of . . . xvi 9

redness in . . . xvi 3, 19

symptoms of . . . xvi 8, 25

a double organ . . . xx 2

affection, in fever, nature of . xvi 234

preventing . . . xvi 235

treatment of . . . xvi 237

of, from circulation . xv 421

affections and gastritis, diagnosis of xix 26

of, in Bright's disease . x 318

of, from lead . . . x 454

of, in heart disease . . xx 262

anatomy of, report on . . xxii 278

ancient physiology of . . xv 451

and alcohol, affinity between viii 540, 541

cerebellum, structure of . vii 504

heart, relation of diseases of xxii 408

kidneys, disease of . . ix 370

liver, sympathy between . ii 140

meninges, sliced, yet cured xv 177

nerves, experiments on by Meyer i 556

offices of . . . xviii 532

nervous centres, Dr. Todd on xxi 452

pia mater, adhesions of . xvi 5

rectum, different heat of . xii 140

spinal cord, action of . v 523

connexion of . xviii 426, 438

injuries of . . iii 10

stomach, relation between ii 370

an excitor of reflex acts . . xix 303

anterior, danger of wounds of xv 178

arrangement of fibres of . . xviii 142

atrophy of . . . . xvi 23

in children . . . xxi 389

average weight of . . . vii 143

bleeding in affections of . . viii 489

in diseases of . . . xxi 103

cancer of the . . . xxii 309

case of cysticercus in . . xx 84

fatal stab of . . . xiii 240

tubercles in . . . vi 200

tumour of . . . vi 226

ulceration of . . iii 549, viii 275

cases of disease of . . . xxiv 323

severe lesion of . . v 236

tumours in . . . iv 376

cellular infiltration in . . xvi 20

changes of, in typhoid fever . ii 50

chemical analysis of . . i 288

composition of . . xii 563

chronic softening of, diagnosis of xvi 28

duration of . . . xvi 28

seats of . . . xvi 24

ciliary motions in . . . iii 501

circular fibres of . . . xviii 437

coagulation of blood in vessels of xvii 487

coloured substance of, its use . ii 135

component parts of . . xxiv 577

compressed, on convulsions in xv 171

Brain, compressed, paralysis in . xv 170
compressibility of . . xv 169
compression of, effects of . x 360
Mr. Guthrie on . . xv 169
on stertor in . . iii 251
concussion of, case of, in India xix 76
fact relative to . . iii 11
Mr. Guthrie on . . xv 163
remarks on . . iv 144, v 173
condition of, in sleep . . v 386
congestion of the . . . iv 436
symptoms of . . . xv 420
in softening . . . xvi 6
in children . . . xxi 382
conversion of alkaloids into . xiv 518
convolutions of, M. Foville on xviii 432
decussation of fibres in . . xv 231
deficient excitement of . . iv 431
determination of blood to . xxii 415
development of . . . vii 222
in ovum . . . . ix 18
diagnosis of functional disease of iv 433
different colours in . . vi 413
dimensions of, report on . xxii 278
diminished excitability of . iv 434
disease of, from disease of heart x 286
use of a seton in . . xiv 252
diseases of, in animals . . xxiv 89
in the army and navy xvii 218, 222
in British troops . . ix 69
in United States . . xvi 43
long issue in . . ii 596
disorganization of, case of . v 236
Dr. Abercrombie on diseases of iii 1, 301
Dr. Brigham on the . . xii 520
Dr. Calori on the structure of v 220
Dr. Philip on affections of . ii 129
Dr. Sims on serous effusion in iii 310, 311
effect of removal of . . i 244
effects of disturbed . . iv 62
mental emotion on . . iv 437
opium on . . . xx 19
thought on . . . iv 437
European, measurement of viii 379, 382
excitement of in fever, respiration in xvi 236
exhaustion of, symptoms of . xv 420
exploration of . . . vi 143
exposed during sleep, case of . i 341
Sir A. Cooper's case of . i 340
exsanguine state of . . iv 438
extravasated blood in, cases of xxii 419
extravasation of blood on . iii 320
fibres of, structure of . . xviii 141
fissure of Sylvius in . . xviii 435
French, smaller than British . viii 383
functional diseases of . . iv 427
with cardiac diseases . xxii 424
functions of . . . . x 554
Galen's description of . . xv 451
general decay of . . xv 418, 420
gray substance of . . . vi 400
hemorrhage into, in children . xvii 553
hemorrhagic cavities in . . xvi 20

Brain, hernia of, Mr. Guthrie on . xv 178
how blood may compress the . iii 324
hydatids of, account of . xx 408
hygrometric quality of the . xi 153
hyperemia of, in old age . . xvii 108
hyperesthesia of . . . xvii 418
hypertrophy and atrophy of . iii 317
case of . . xx 229, xxi 387
in children . xx 555, xxi 386
remarks on . . . iii 317
increased excitement of . iv 429
inflammation of, bleeding in . xiv 5
Dr. Hope on . . . xii 100
forms of . . . iii 305-7
in children . . . xxi 385
on mercury in . . xii 102
inflammatory affections of . xxii 384
irritation of, treatment of xx 36
influenced by pericardium . xii 333
injuries of head affecting . xv 161
injuries of, not painful . . v 523-5
injury of, incredible case of . vii 585
intimate structure of . . vi 393, 400
in vertebrata, on fusion of parts in xxii 494
irritable, symptoms of . . xv 421
irritation of, effects of . . iv 430
its influence on the heart . ii 132
its special functions . . xxii 516
localization of injuries of . xv 162
malformation of, case of . . xxiv 323
measurement of Negro . viii 379, 382
mental physiology of . . ix 222
metastasis of rheumatism to . xxii 427
morbid arteriality of . . iv 438
excitability of . . . iv 432
venosity of . . . iv 438
movements after removing . v 514
M. Foville on anatomy of xviii 423, 432
Mr. Solly's work on . iv 485, xxiv 572
M. S. Pinel on pathology of . xx 18
nervous debility from overtasked xv 420
non-congestion of, in hanging xxii 412
œdema of, M. Pinel on . . xx 23
of birds, peculiarities of . . viii 228
of childhood, on overworking the iii 474
of children, pathology of . viii 489
weight of . . . xxi 387
of Europeans, weight of . . viii 375
of fishes, observations on . xxii 495
on homology of . . xxiv 573
of man and animals, Harlan on ii 518
of Negro, discussion on . . v 585
and orang-outang . . viii 381
on the size of . . . iv 529
weight of . . viii 377, 382
of the insane, atrophy of . ii 461
operations of the . . . xvi 159
organic diseases of . . . iii 315
ossification in softening of . xvi 31
over-exertion of, remarks on . i 340
overtasking the, remarks on . iv 56
pain of the . . . . xvii 418
paracentesis of, case of . . iii 311
Brain, particular attribute of the . xxiv 185
peduncular fibres of . . xviii 436
perforated spot in . xviii 435, 437
spot of, structure of . xviii 437
period of greatest size of . viii 376
perverted nutrition of . . iii 312
physiology of . . xvii 265, xxii 488
remarks on . . . xviii 145
Unzer on . . . xxiv 185
plethora of . . . . iv 437
plurality of organs in . . ix 197
posterior lobe of, wanting in lower mammalia . . xxii 502
pressure on, on coma from . xxii 405
proportion of, to the body . viii 376
pseudo-morbid appearances of xiii 562
quantity of blood in, unvaried xii 99, 100
ramollissement of . . . iii 312
rapidity of growth of . . xvii 371
red softening of . . . iii 312
reflex function of . . . xix 298
report on . . . . xix 578
researches on softening of . x 259
sanguineous infiltration of . xvi 6
seat of affections of . . vi 145
sensibility to pain of . . iv 431
sieve-like condition of . . xiv 226
size of . . . . . . xvii 265
a measure of mental power ix 198, 201
as a measure of power in v 586
softened, anatomy of . . xi 464
febrile stage of . . xi 468, 470
on the colour of . . xi 464
paralytic stage of . . xi 467, 469
premonitory stage of . xi 466, 469
softening of xi 464, xiii 535, xiv 547, xx 228
absurd theory of . . xvi 32
actual pain in . . . xvi 27
acute . . . . . xvi 1
a form of gangrene . . xi 472
apoplectiform . . xvi 9, 15
case of . . . . xi 472
causes of . . . xi 465, 471
cephalalgia in . . xvi 11, 27
changes in . . . xvi 20
chronic . . . . xvi 18, 27
chronic, pulpy . . xvi 19, 29
contraction in . xvi 10, 16, 26
convulsions in . . xvi 10, 16
curability of . iii 313, xvi 29
cold in . . . . xvi 11
diagnosis of . vi 144, xi 470, xvi 14
extent of . . . xi 465
forms of . . . xvi 4
gangrenous . . . xvi 31
in children . . . xxi 390
intellect in . . . xvi 12
in the young . . . xi 471
movement in . . . xvi 10, 26
M. Pinel on . . . xx 35
softening of, nature of . xvi 31
paralysis in . . . xvi 10
prognosis of . . . xvi 30

Brain, softening of, rigidity of limbs in iii 313

sensation in . . . xvi 10

sensibility in . . . xvi 11, 26

supposed cure of . . xxii 462

symptoms of . . . xiv 548

the face in . . . xvi 12

the heart in . . . xvi 33

the pulse in . . . xvi 13

the pupils in . . . xvi 13

the speech in . . . xvi 27

the tongue in . . . xvi 26

transient palsy in . . xxii 406

treatise on . . . xvi 1

treatment in . . . xvi 33

vascularity in . . . xvi 8

yellow tint in . . . xvi 3, 19

spinal cord and sympathetic ganglia, analogy of . . xxiv 203

state of, in concussion . . iv 439

structure of . . . x 296, xxiv 130

cortical substance of . x 253

Dr. Macartney on . ii 134

study of, by comparative anatomy xxii 489

principle in . . . xxiv 577

substance of, tubular . . vi 401

suppuration of, case of . . v 174

sympathetic action of, on liver xii 83

diseases from . . . xxii 406

symptoms of organic disease of iii 316

table of injuries of . . . xii 70

termination of fibres in . . v 300

the human, Mr. Solly on . xxiv 572

the most vital part of . . i 560

the organ of the mind . . ix 197

Theophilus' hypothesis on . xv 452

the source of vital power . i 558

torpor of the, causes of . . iv 435

effects and signs of . iv 424

tubercles in . . . . ix 574

in children . xv 152, xvii 552

case of . . . . xiii 503

tubercular disease of . . . iii 315

tubules of, contents of . . vi 403

organization of . . vi 403

walls of . . . . vi 404

tubulo-fibrous structure of . iii 547

ulceration of . . . . xvi 23

unaffected by stimulants . v 523

varicose or jointed tubes of . vi 401

vascular disease of, effects of . iii 326

venous reflux in . . . xxii 413

vessels of, how protected . xxiii 142

relative fulness of . . xvii 127

unalterable fulness of . xvii 127

weight of . . . . xvii 265

its relation to age . . iii 318

altered by diseases . . viii 375

Negro and European . v 585

table of . . . . iii 318

white substance of . . vi 400

white softening of . . . xvi 32

yellow softening of . . iii 312

Brainular, an objectionable term . iii 198

Braithwaite, Mr., his retrospect xi 224

Bramley, Mr., on bronchocele viii 103, 116

on the bronchocele of Nepâl . iii 354

Branchlets, from leaf of a plant . xxiv 437

Brandis, Dr., his therapeutical doctrines ii 530

on use of cold in disease . ii 529

Brazilian quartz, or pebble lenses . xvi 362

Bread-and-water breakfast . . xvii 47

diet, effects of . iv 192, xiii 492

effects of living on . . xvii 28

Bread, brown, fresh, poisoning by . xviii 556

Dodson's unfermented . . xiii 489

fermented, loss of sugar in . xiii 490

gluten, M. Bouchardat's . xvi 484

injurious effects of alum in . xvii 25

loss of gluten in fermenting . xiii 489

mouldy, on poisoning by . xviii 555

on noxious ingredients in . xviii 556

pills, efficacy of, in diarrhœa . xxi 249

unfermented, Dr. Thomson's . xiii 491

unleavened, use of, by Jews . xiii 491

use of, in India . . xiii 491

use of, in Scotland . . xiii 491

Breakfast of bread and water . xvii 47

Breast, absorbents of the . . x 119

arteries and veins of . . x 118

cancer of, cause of frequency of xxii 159

comparative anatomy of . . x 128

deep-seated abscess of . . xiii 264

evolution of . . . . x 122

fascia of . . . . . x 115

fat of the, use of . . . x 118

female, Mr. Tuson on . xxii 173

sero-cystic tumours of . xxii 167

fungous tumours of, case of . xxii 157

inflammation of, iodine in . viii 521

irritable, remedy for . . vii 530

meal, formula for . . . xiii 87

nerves of . . . . . x 119

on neuralgia of . . . xxii 158

sero-cystic tumours of . . x 587

Sir A. Cooper on . . ix 538, x 101

treatise on diseases of . . xxii 156

Breasts and genitals, sympathy between x 111

component parts of . . x 108

excoriated, remedy for . . v 575

influence of gestation on . x 123

instance of four, in a female . x 109

position of . . . . x 106, 108

streaks in, during pregnancy . iv 465

Breath, affected by food and drink . i 242

by hunger . . . i 246

by pregnancy . . . i 246

condensed, organic matter in . xxiv 256

difference of, asleep and awake i 242

experiments on . . . i 243

fetid, blundered case of . . v 139

in bronchitic cases . . xvii 112

in putrid diseases . . i 246

offensive, in starvation . . i 246

often offensive in old age . i 246

on oxydation of the blood by . xvii 153

sour, in rickets and scrofula . i 246

Breath, tainted during catamenia . i 246

urinous in ischuria . . i 246

volatile substances detected in i 243

volume of the . . . xvii 153

Breathing, capacity of . . . xvii 256

of child during birth . . iv 101

manner of, important in diagnosis i 481

Breech case, with fractured thighs . xiv 256

presentations, diagnosis of . ii 78

Dr. Collins on . . ii 78

Dr. Meigs on . . viii 47

in 16,434 labours . . ii 72

management of . . iii 407

Breeding, communication of sex in . vii 380

of animals, Mr. Walker on . vii 381

Breeds, remarks on crossing . . iii 373

Bremberg, Dr., a quack in Russia, account of . . . . . i 600

Brera, Prof., on a lithotriptic water ix 246

on the ballota lanata . . i 566

Brereton, Mr., his clinical reports xxiii 421

Breschet, M., on episternal bones viii 248

on rabies . . . . . xi 229

on temperature of the body . i 246

on the breath . . . . i 243

on the lymphatics, remarks on iv 349

on the lymphatic system . iv 325

on the organ of hearing . viii 68-9

on the structure of the skin . ii 429

Breslau, medical school of . . i 299

university of, account of . i 293

Brett, Mr., his plagiarisms of Dr. Mackenzie . . xxiv 365, 370

on cataract, &c. . . . . xxiv 364

on surgery in India . . xii 422

Brewer, Dr., on spinal deformity xvi 347

specimen of his English . xvi 352

Brewster, Sir D., on cataract . v 602

on crystalline lenses . . ii 218

Brickmaking by teetotallers . . xxiv 535

Bridewell and Bethlem hospitals . xx 471

Bridgewater treatises, defect in the xxi 116

Bridgman, Laura, allusion to her case xxiv 478

Brigham, Dr., on consumption in America . . . . . iii 526

on religion and education . iv 55

on the brain, &c. . . . xii 520

Bright and Addison, Drs., on the practice of medicine . ix 461, 494

Bright, Dr., commendation of his writings . . . . . iii 157

great value of his cases . . iv 380

his practice of medicine . iii 452

on abdominal tumours vi 72, vii 462, 470

on cerebral tumours . . iv 376

on continued fever . . xii 313

on diagnosis of peritoneal adhesions . . . . . ii 167

on electricity as a remedy . vi 72

on jaundice . . . . iii 157

on organic change of kidneys ii 223

on renal disease . . . x 301

on spasmodic diseases . . ix 431

Bright's disease, acute form of . x 308

hæmaturia in . . x 323

albuminous urine in . viii 153

albuminuria in x 311, 322, xiii 443

on anasarca in . x 313, 325

anatomical characters of x 305

blood in . . xiii 440, 442

cardiac disease with . xiii 439

cause of dropsy in . xiii 442

changes of the blood in . xvii 151

coma in . . . x 318

complications of . . xiii 439

cylinders in urine in . xxiii 504

diagnosis of . x 314, 321, 323

diaphoretics in . . x 327

diarrhœa in . . . x 318

on different names for . viii 133

diseases produced by . xxiv 571

diuretics in . . . x 328

does it exist? . . xiii 560

Dr. Johnson on . . xxiv 321

duration of . . . x 320

effect of on blood . . xix 32

eight varieties of . . xv 100

on tartar emetic in . x 327

etiology of . . x 320, xiii 442

from lithotomy . . x 315

frequency of . x 323, xiii 443

gradations of . . x 306

granulations in . . x 324

history of . . . x 304

in infants . . . xiii 444

inflammatory . . xv 100

inflammatory or not . x 324

in pregnancy . . . x 319

Malpighi's glands in . xiii 436

M. Becquerel on . . xiii 435

morbid anatomy of . xv 100

Mr. Toynbee on . . xxiv 332

pericarditis from . . xxiv 569

phases or stages of . xiii 436

plausible theory of . xiii 438

prognosis in . . . x 325

salts of the serum in . xvii 151

renal discoloration in . xiii 436

state of blood in . x 312, xviii 336

table of urine in . . xiii 441

treatment in acute . x 326

urine in . . . xiii 440

in acute . . x 308, 322

in chronic . . x 311, 322

with bronchitis . . x 317

with cardiac affections . x 316

with phthisis . . . x 317

Brighton, and its three climates . xvi 500

affection of the eyes at . . xvi 502

bad for congestive diseases . xvi 502

inflammatory dyspepsia . xvi 502

choice of a residence at . . xvi 501

illness from the Chain Pier at xvi 502

months most salubrious in . xvi 501

pure air of the Downs at . xvi 502

topography of . . . xvi 500

Bristol and Worcester, mortality of . vi 6
medical topography of . . vi 10
sanitary condition of . . xxi 352
water of . . . . . xiv 352
Briquet, Dr., on mercury in smallpox ix 249
Britain, exhaustion of the fields of xvii 226
British and Foreign Medical Review,
postscript to . . xxiv 585
expenditure and income of xxiv 596
British Association, committee of . v 589
meeting of . . . iv 567
reports of . . . i 368
for 1836, report of . ii 594
report of . . . vi 573
British journals, list of papers in . xv 575
medical papers in . . xi 534
original papers in . . i 309, 615
British legion, causes of sickness in vi 449
in Spain, history of . vi 448
mortality in . . vi 451, 454
type of fever in . . vi 450
British medical corporations, statistics of i 306
journals, list of . . i 223
original papers in . xv 256
British physicians, over-activity of ix 465
British statesmen, responsibility of xiv 454
British troops, mortality of, abroad iii 49
Brittan, Dr., his translation of
Malgaigne . . . xxiii 155
Brittleness of bones, hereditary . xxii 49
Broc, M., on teaching anatomy . xiii 519
Brodie, Sir B., his case of tracheotomy xviii 370
his lectures on surgery . . xxii 160
his Hunterian oration, 1837 . iv 189
his position and works . . xiv 159
on diseases of joints . . x 330
local nervous diseases . xii 121
medical education . . xxiii 242
nervous affections . . iv 132
pulmonary exhalation . i 241
duties of students . . xvii 188
the spinal cord . . iv 143
urinary organs . . . xiv 159
watery vapour from the lungs i 241
opinions of . . . . xvii 188
Broiling of food, remarks on . xvii 37
Bronchial affections, climates for . xii 163
calculus, case of . . . xviii 365
coagula, ramifying, figure of . xxiii 505
thallus-filaments in . xxiii 510
discharges, thermal baths in . xiv 340
diseases, causes of . . vi 25
inflammation in . iv 298, 300
mineral waters in . . xiv 341
Sir J. Clark on . . xiv 341
glands, blackening of the . i 137
disease of . . . xx 252
phthisis, diagnosis of . . xvii 309
in children . . xvii 305, xx 39
symptomatology of . xvii 308
polypus . . . . . vi 268
Professor Casper on . ii 554
respiration in children . . xii 358
Bronchial respiration, on normal . ix 306
tuberculization . . . xvi 435
tubes, dilatation of . . xi 403
secretions from . . xi 404
terminations of . . xi 405
Bronchi, albuminous exudation in . xii 109
application of caustic to . xxiv 503
case of spike of oat in . . vii 251
dilated, diagnosis of . iv 310, xi 404
complications of . . . iv 310
dilatation of iv 309, 311, xiii 386, xv 85, 558
causes of . . . xi 404
in phthisis . . . xxiii 435
Laennec on . . . iv 312
origin of . . . xv 85
foreign bodies in . . v 440, xx 245
frequency of false membrane in xii 109
irritation of, secretions in . iv 300
minute, mode of showing . xi 405
morbid anatomy of . . xi 402
nature of affections of . . iv 298
never anastomose . . . iv 308
obliteration of . . . iv 309
obstruction of . . . iv 308, 311
secretions in affections of . iv 299
smaller, sibilant sound in . i 482
tuberculous disease of . . xv 86
false membrane in . . xvi 429
terminations of . . xvii 94, xxiii 430
tubercular perforation of . xvii 306
expectoration from . . xx 96
ultimate distribution of . . xiv 573
Bronchitis, acute, point of danger in ix 488
primary . . . . iv 300
secondary, in fevers . iv 303
and pneumonia distinct . . iv 314
and tubercle, Dr. Otto on . ix 217
antimonial mixture for . . iv 306
bituminous medicines in . xii 432
on bloodletting in . . . iv 305
burning heat of skin in . . vi 136
capillary, M. Flauvel on . . xix 395
strychnia in . . . xx 444
cause of death in . . . ix 488
chlorine inhalations in . . iv 224
chronic, and phthisis . . xxiv 505
cathartics in . . . xvi 245
emetics in . . . xvi 246
secondary . . . iv 304
sulphur in . . . xvi 246
treatment of . . xvi 245, xx 448
counter-irritant for . . iv 308
diagnosis of . . . . xx 443
from phthisis . . iv 320
Dr. Graves on . . . xvi 244
Dr. Stokes on . . . iv 298
division of . . . iv 299
dry, copaiba and cubebs in . ii 229
emetics in early stage of . xx 245
emetic tartar in . . . i 421
epidemic, account of . . xx 245
expectorations in . . . iv 301
false membrane in . . . xii 356

Bronchitis, full bleeding in . . ix 488

general capillary . . . xx 442

in burns and scalds . . v 440

in children, prognosis in . xii 360

secretions in . . . xii 356

treatises on . . . xii 150

treatment . . . xii 361

in dentition, Dr. Stokes on . iv 299

inspiration of cold air in . xx 449

in typhus, treatment of . . iv 308

microscopic anatomy of . . xix 109

more common in the male than female . . . . . i 404

M. Raciborski on sound in . i 487

M. Valleix on . . . . xx 441

observations on (Append. to vol.) xi 36

of children, sounds in . . xii 357

works on . . . xii 350

organic changes in . . iv 308

physical diagnosis of . . iv 302

signs of . . . . iv 301

plastic . . . . . . x 286

relation of, to croup . . xii 356

relative prevalence of . . vi 24

scrofulous, mercury in . . ix 492

secondary, engorged lung in . iv 303

secretion of pus in . . iv 301, 316

sibilant rhonchus in . . i 487

sound of respiration in . . iv 302

stethoscopic signs in . . xvi 245

stimulants in . . . iv 307-8

stimulating mixture for . . iv 307

summer, or hay fever . . xx 450

tartar emetic in . . . iv 306

treatment of iv 305, 307, xvi 245, xx 443

use of acetate of lead in . . x 288

chlorine in . . . vi 533

vesicular in children . . xii 354

with disease of kidneys . . x 317

syphilitic affections . iv 304

Bronchocele, cause of . . viii 106, xx 174

encysted, case of . . . vii 586

endemic, treatise on . . xx 168

general remarks on . . viii 103

Himalayan, treatise on . . iii 354

in a fœtus . . . . xi 229

Indian ointment for . . xix 74

in relation to limestone formation iii 354

not from snow-water . . viii 104

of Nepâl, Mr. Bramley on the . iii 354

operation for . . . xxii 50

origin of . . . . xii 119

relative prevalence of . . vi 27

Sir A. Carlisle on . . . vi 188

treatises on . . . . viii 103

treatment of . . . . viii 119

in India . . . xix 74

tying the arteries in . . ii 260

use of iodine in . . . viii 119

Bronchophony, absent in typhoid pneumonia . . . i 488

in pleurisy, remarks on . . xiv 177

not always in pneumonia . i 487

Bronchophony, observations on . xiv 175

slight, natural . . . ix 308

suspension of, by mucus . xiv 176

Bronchoplastic, etymology of . vii 411

operation . . . . . vii 411

Bronchotomy, cases of . . . v 430

chances of success in . . xv 327

diseases requiring . . . xxi 488

observations on . . v 441, xii 110

remarkable case of . . xii 256

Bronchorrœa, chlorine inhalations in iv 224

use of creosote in . . . ii 169

Bronchus, extraction of a button from xxii 376

foreign body in, case of . xviii 370

removed from, by operation v 466

Broom-seed, its use in dropsy . i 533

preparation and tincture of . i 534

Broom, seed of, preferable to its leaves and roots . . i 533

Broths and soups, constituents of . xvii 22

Brougham, Lord, his error in statistics iii 52

on honeycombs . . . xi 97

Broussais compared with Clutterbuck v 185

and his doctrines, account of iii 388, 390

and Pinel, war between . . ii 387-8

and Prost compared . . xii 296

anticipated by Prost . . xii 296

bitterness of his opponents . iii 388

decline of his reputation . iii 448

his doctrine founded on irritation ii 3

his influence on French medicine iii 390

in his chair, picture of . . iii 448

list of his works . . . vii 297

mortality among his patients . ii 5

on epidemic meningitis . . xxiii 82

on phrenology . . . ix 190

obituary of . . . . vii 297

profane title given to . . iii 391

remarks on his doctrines . vii 297

zeal of his partisans . . iii 388

Broussaisian doctrine in Florence and

Rome . . . . . i 497

practice, sad picture of . iii 389

Browne, Dr. W. A. F., on bleeding in mania . . . . xi 543

his circuitous oratory . . v 71, 73

on definitions of insanity . v 66

on lunatic asylums . . v 65

opposite assertions of . . v 65

Brown, Mr. A., on glanders in man iv 255

Brown, Mr. J. B., on scarlatina . xxi 497

Brown, on his theory of excitement v 80

Brown, Sir Thomas, remarks on . xix 123

Brownism, Italian . . . vii 424

Bruce, Robert, condition of his bones v 541

Brucia, distinction of, from strychnia xvi 79

process for detecting . . xvi 79

Bruckenau and Bocklet, waters of . xiv 355

Bruit de diable, Dr. Ward on iv 245, vi 392

remarks on the . . v 427

de frottement in pericarditis . xii 113

de mouche, cause of the . v 427

de soufflet, in endocarditis . ii 327-8

Bruit de soufflet, its nature . . i 451
mechanism of . iii 257, vii 276
ou ronflement du diable . i 449
why so termed . i 456
Brunel, Mr., account of his case . xviii 370
Brunner's glands, anatomy of . i 527
diseases of . . . . xi 420
Brunonian system, Dr. Knolz on . xix 371
Bruns, Dr., his general anatomy xiv 478 480
Brussels, increasing destitution in . xix 132
mortality of . . . xix 126, 128
population of, statistics of . xix 128
state of the poor districts in . xix 127
Bruta, fossil, on the teeth of . . xxi 70
Brutality, Dr. Mayo on . . . xxiv 232
Brute pathology, observations on . xxii 35
Brutes, scrofula frequent in . . xxiii 518
Bubbles, commotion caused by . xiv 317
Bubo, venereal, nature of . . v 11, 29
new mode of treating . x 418
Buboes and boils, use of iodine in . viii 521
nature and diagnosis of . . x 386
on inoculation from . . x 387
Bubon d'emblée, of French writers. x 386
Buchanan, Mr., his case of cancer cured . . . . . iii 557
Buckingham, Mr., his bill for public walks, &c. . . . i 304
Bucnemia tropica of India, account of iii 353
scrotal form of . . iii 353
Budd, Dr. W., his retrospective address xx 509
on movements in palsy . . ix 437
on symmetrical disease . . xv 149
Budd, Dr. G., on diseases of the liver xxi 39
on emphysema of lungs . . xi 448
Bude light, principle of the . . xix 18
Budge, Dr., his experiments. . xvii 400
on the nervous system . . xvii 379
on vomiting . . . . . xv 60, 74
Buds and young shoots as food . xvii 20
Bufalini, Professor, on clinical medicine . . . . . xxiv 360
Buffon, his errors on animal gestation . . . . . . ii 403
Buffy coat, composed of oxyprotein xviii 263
Mulder's researches on the xvii 431
of blood, Dr. Williams on the xvii 481
formation of xiv 589, xvii 250
in inflammation . xvii 147
in pregnancy . . xvii 148
microscopy of . . xxiii 507
peculiar state of . xiv 553
remarks on . . viii 347
on the blood during pregnancy iv 459
remarks on . . vi 136
on chlorotic blood . . xvii 141
on horse's blood, normal . xvii 136
Bugliarelli, Dr., his remedy for dartres . . . . . iii 241
Buhlmann, M., on the sputa . xvii 519
Buildings in towns, regulations for xxi 344
Bulard, M., on the oriental plague xvi 289
Bulard, Dr., on the plague . v 560, viii 550
Bulb of urethra, anatomy of . . xix 508
Bulbo-cavernosus muscle, action of xix 511
Bulbus vestibuli of the vagina . xix 514
Bullæ, description of . . . xv 385
or bullous inflammation . . ii 149
syphilitic . . . . . xxiii 355
Bullar, Dr., on the Azores . . xii 224
Bull, Cæsarean operation by a . iii 248
Bull, Dr., his hints to mothers . v 215
on the management of children xi 224
Bulletin of the medical sciences . i 547
of insane at Charenton . . i 273
Bulwer, J. B., Philocophus by . viii 69, 71
Bunions and corns, treatise on . xix 560
and ganglions, Mr. Key on . iii 151-2
cause of in weakly girls . . iii 153
Burchard, Dr., on tumours of the scalp . . . . vii 74
Burdach, M., on the nerves . vi 394, 409
his summary . . . vi 410
Burder, Dr., life and character of xvii 289
Burder, Dr. T. H., obituary of . xvii 289
Burdin and Dubois, MM., on animal magnetism . . . xix 428
Burgess, Dr., his physiology of blushing . . . . viii 245
on diseases of the skin . xv 372, 399
Burggraeve, M., his textural anatomy . . . . . . xxiii 136
Burial grounds, effects of, on health xv 513
extent of . . . xv 521
in London, state of . x 214
observations on . xv 520, 522
in towns, Dr. Reid on . xix 4
places among the living, evils of x 212
societies, incentive to murder xvii 405
Burials, clergyman's fees at . . xv 513
Burke, Mr., on fractures . . vi 308
Burking, antiquity of . . . ix 50
Burmese empire, medical reports on xv 1
Burn, remarkable case of . . vii 287
extensive, remarkable case of xix 247
Burne, Dr., on diseases of the cæcum ix 445
on habitual constipation . . x 40, 48
on hydrophobia . . . vi 268
on inflamed cæcum . . iv 148
Burnes, Dr., his case of fork in the back . . . . . v 609
Burnett, Mr., on the Divine attributes vii 227
Burnett, Sir W., his discovery . xi 533
inaccuracy respecting . . xii 94
obligations of navy to . . xi 182
Burning of the feet, an Indian disease v 131
description of. . . v 132
in spirit-drinkers . . v 132
morbid anatomy of. . v 132
treatment of . . v 132
the crown of the head, use of vii 63
Burns, adhesive straps how applied to i 580
and scalds, M. Vidal on . . xi 311
use of iodine in . . viii 522

Burns, bleeding in . . . xi 314

causes of death from . . xi 313

chloride of soda in, Lisfranc on xiv 19

cicatrices from, operations on xix 410

classification of . . . xiv 18

contractions from . . xi 315, xiv 20

deformity from, cases of. . xviii 525

Dr. Mutter on . xviii 525

extensive, on coma from . xviii 217

four periods of danger in . xiv 18

ice to the head recommended in i 496

M. Lisfranc on . . . xiv 18

on treatment of . . xiv 18

new mode of treating . . vii 287

new treatment of, by Prof. Velpeau i 579

objection to cotton in . . xi 314

of the eye, from gunpowder . ii 267

prognosis in . . . . xi 313

six degrees of . . . xi 312

specifics for all . . . xiv 18

three stages in . . . xi 313

topical applications to . . xi 314

treated by yellow wash . i 581

treatment of . . . . xi 313

by cold water . . iii 119

in France . . . i 496

ulceration of duodenum in . xv 155

use of pressure in . . . xi 314

various treatment of . . i 580

Burns, Dr., on dilating os uteri, error of ii 84

on reunion of parts . . vii 434

Burnt holes, disease of, account of xv 387

Burrows, Dr., on cerebral disorders xxii 408

on carcinoma of the lungs . xx 90

Burrowes, Dr., on measles and scarlatina . . . . . xii 98

Bursæ, inflamed, use of iodine in . viii 523

Bursal tumour, iodine injection in xviii 89

Burton, Dr., on the gums . . xi 450

Burying in churches, danger of . x 213

in towns, laws against . xv 516

Bush, Dr., his Berlin Midwifery Report . . . . . v 576

Bushe, Dr., on diseases of the rectum iv 467

Bushe, Mr., on the operation for cleft palate . . . ii 493

Bushmen of South Africa, origin of xxiv 77

Bushnan, Dr., on dysmenorrhœa . x 593

on instinct and reason . . xi 90, 103

Busk, Mr., case of deficient kidney by xxiv 329

on abscess . . . . xxiv 330

Bute, Isle of, its climate . . xii 168

Butchers, healthiness of (App. to vol.) xi 39

Butler, Mr. C., reminiscences of xviii 234

Butterflies, much uric acid in . xix 570

Buttermilk fatal to the horse . . iv 130

Buttock-hump of the Hottentots xxiv 451

Button farcy . . . . xxii 48

Butyric acid a product of fibrin . xix 250

Buxton baths, effects of . . . v 397

water of . . . . . xiv 352

Buzorini, Dr., on typhus fever viii 428, 442

Bygone epidemics, relics of . . v 193

Cabanis, his philosophy . . v 83

Cabbage, nutritious properties of . xiii 492

raw, on its digestibility . ii 387

Cabot, S., account of his expedition i 599

Cachectic diseases in animals . xxiv 87

Cachexiæ, classification of, by Mr. Travers ii 20

Dr. Bene on the . . . i 16

Cachexia, Londinensis of Sir J. Clark xi 61

mercurial, signs of . . ii 21

Mr. Travers on . . . ii 19

syphilitic, signs of . . ii 20

splenic, description of . . iii 345

African, Dr. Ferguson on . iii 217

symptoms of . . iii 217

tuberculous, Sir J. Clark on . i 72, 74

venous, active and torpid . xx 431

Caduca, formation of the . . ix 25

Cæcal digestion, remarks on . xvi 217, 224

fistula, large in loins . . xi 248

Cæci appendix, perforation of . iv 149

Cæconitis, a wrong term, by Dr. F. W. Smith . . . . i 506

Cæcum, acute inflammation of . ix 449

appendix of, foreign bodies in ix 453

cases of obstructed . . ix 450

chronic diseases of . . iv 149

collections of pus in . . ix 451

digestive action in . . xvi 149

Dr. Albers on diseases of . ix 445

Dr. Burne on diseases of . ix 445

fæcal, tumours of . . . ix 446, 447

French writers on disease of . i 503

gastrotomy in disease of . ix 250

hernial state of . . . xxii 195

inflamed, cold-water douche in i 506

diagnosis of . . . iv 148-9

treatment of . . . i 506

peculiarities of . . iv 148

symptoms of . . iv 148-9

inflammation of . . . ix 446, 448

cause of . . . i 504

described . . i 504, xviii 312

in dysentery . . . iii 334

insidious . . . i 504

symptoms of . . . i 504

three kinds of. . . i 504

lactic and butyric acids in . xvi 149

obstructions of . . . ix 450

perforation in abscess of . ix 451

of appendix of . . ix 452

rupture of during labour . v 256

the seat of a second digestion . v 402

tumours of, treatment of . ix 457

Unger, Ferrall, and Smith, on i 501

Cæsalpinia Bonducella in ague . iii 348

Cæsarean operation, accidental . iii 248

cases of . . . ii 569, xii 261

doubly successful . . xi 530

description of a . . ii 270

limit requiring . . viii 52

Professor Kilian on . . iii 422

successful . . . xiii 545

section, bad success of . . xiv 367

